# Biochemical Characterization of Human Retroviral-Like Aspartic Protease 1 (ASPRV1)

**DOI:** 10.3390/biom10071004

**Published:** 2020-07-06

**Authors:** Mária Golda, János András Mótyán, Katalin Nagy, Krisztina Matúz, Tibor Nagy, József Tőzsér

**Affiliations:** 1 Department of Biochemistry and Molecular Biology, Faculty of Medicine, University of Debrecen, 4032 Debrecen, Hungary; golda.maria@med.unideb.hu (M.G.); 88.nagy.kat@gmail.com (K.N.); matuz.krisztina@med.unideb.hu (K.M.); 2 Doctoral School of Molecular Cell and Immune Biology, University of Debrecen, 4032 Debrecen, Hungary; 3 Department of Applied Chemistry, Faculty of Science and Technology, University of Debrecen, 4032 Debrecen, Hungary; nagy.tibor@science.unideb.hu

**Keywords:** ASPRV1, SASPase, protease, retroviral-like protease, retroviral-like aspartic protease 1, skin-specific aspartic protease, homology modeling, protease inhibitor

## Abstract

The human retroviral-like aspartic protease 1 (ASPRV1) is a mammalian retroviral-like enzyme that catalyzes a critical proteolytic step during epidermal differentiation; therefore, it is also referred to as skin-specific aspartic protease (SASPase). Neutrophil granulocytes were also found recently to express ASPRV1 that is involved in the progression of acute chronic inflammation of the central nervous system, especially in autoimmune encephalomyelitis. Thus, investigation of ASPRV1 is important due to its therapeutic or diagnostic potential. We investigated the structural characteristics of ASPRV1 by homology modeling; analysis of the proposed structure was used for interpretation of in vitro specificity studies. For in-vitro characterization, activities of SASP28 and SASP14 enzyme forms were measured using synthetic oligopeptide substrates. We demonstrated that self-processing of SASP28 precursor causes autoactivation of the protease. The highest activity was measured for GST-SASP14 at neutral pH and at high ionic strength, and we proved that pepstatin A and acetyl-pepstatin can also inhibit the protease. In agreement with the structural characteristics, the relatively lower urea dissociation constant implied lower dimer stability of SASP14 compared to that of HIV-1 protease. The obtained structural and biochemical characteristics support better understanding of ASPRV1 function in the skin and central nervous system.

## 1. Introduction

The retroviral-like aspartic protease 1 (ASPRV1) is an endogenously expressed mammalian protein. It has been identified as a protease of the human epidermis (EC: 3.4.23.-) that is specifically expressed in the granular layer of the skin, thus it is also referred to as skin-specific aspartic protease (SASPase) [1,2]. The presence of its protease domain implied its proteolytic function, while exhibiting similarities with the retroviral aspartic protease family indicated its retroviral origin [1].

The function of ASPRV1 in the skin is well-studied. In the basal layer of the epidermis, the keratinocytes divide and daughter cells go through differentiation. Keratinocytes show characteristic morphological and biochemical changes while migrating toward upper layers. During their terminal differentiation and particular form of apoptosis, the keratinocytes lose their intracellular organelles and transform into flattened dead cells (corneocytes), which are then eliminated from the skin surface by desquamation [3]. The differentiating keratinocytes express several proteases (PR), which are organized into cascades; the activation of these proteolytic cascades is essential for the maintenance of skin physiology due to proteolytic processing of target proteins [4].

**Figure 1:**
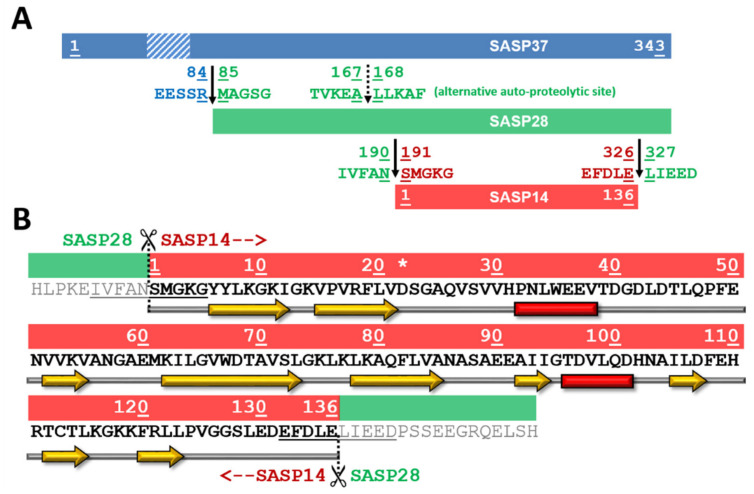
Schematic representation of SASP37, SASP28, and SASP14 forms. (**A**) Schematic representation of SASP37, SASP28, and SASP14 forms is shown. A transmembrane domain (F56-E77) at the N-terminal region of SASP37 is shown by a dashed box based on literature data [1,2]). SASP37 numbering was used to label boundaries of different ASPRV1 forms and processing site sequences. (**B**) Sequence and predicted secondary structure is shown for SASP14, using SASP14 numbering (S1-E136). Asterisk indicates the catalytic aspartate of the consensus active site motif. The N- and C-terminal processing sites are underlined [1]. Predicted secondary structure is also indicated, yellow arrows indicate β-strands, while red boxes indicate α-helices.

ASPRV1 was also found to be part of such a proteolytic cascade. It is specifically expressed in the *stratum granulosum* in its presumably inactive form [1,2] and further processed to shorter forms, which are named after their molecular weights (Figure 1). The full-length protein that contains a putative transmembrane domain has a 37 kDa molecular weight (SASP37). The pro-form is considered to be SASP28 (28 kDa) and its autoprocessing results in the mature SASP14 (14 kDa) that consists of only the protease domain [1].

The natural substrate of the mature SASP14 appears to be profilaggrin (pro-FLG), other natural substrates of ASPRV1 have not been identified to date. Pro-FLG has a >400 kDa molecular weight and consists of filaggrin (FLG) monomer repeats. During its maturation, pro-FLG is cleaved at the GSFLY↓QVST monomer linker sequences (the arrow denotes cleavage position within the sequence), leading to the release of FLG monomers (human FLG monomers have ~37 kDa molecular weight) [5]. The resulting monomers are further modified and degraded to short peptides and amino acids. These degradation products constitute a part of the natural moisturizing factor which plays key role in moisturization and protection of the skin from external environmental factors [5].

Functional studies have already been performed to explore the role of ASPRV1; both sites directed mutagenesis and studies on mice lacking *ASPRV1* gene confirmed the role of ASPRV1 in pro-FLG processing and in the maintenance of normal water conditions of the skin [1,2,5]. The role of ASPRV1 in skin regeneration was also proved using a mouse model [6], and correlations of ASPRV1 with ichthyosis have also been reported; both dog [7] and human [8] variations were found to have role in the development of this skin disorder. These variations affect the protease, a dog variation that corresponds to L325P mutation of human protein affects the P2 residue of the C-terminal cleavage site of the protease domain [7], while K199E, R311P, and P314T variations of human ASPRV1 are in the proximities of N- and C-termini of human SASP14 [8]. These human variants were found to cause loss of protease function; each variant showed impaired autoproteolysis and filaggrin processing [8]. The molecular mechanisms behind the manifestation of symptoms are less well-understood, future studies need to reveal the possible effects of unprocessed ASPRV1 or filaggrin accumulation.

While previous studies focused almost exclusively on the expression and function of ASPRV1 in epidermis, recently ICAM1^+^ macrophage-like neutrophil cells of the mouse and human immune systems were also found to express ASPRV1 [9]. In that study, ASPRV1 mRNA was found to be the most abundant in blood neutrophils compared to other leukocytes, and elevation of its level was detected in the brain lesions of patients suffering from severe multiple sclerosis (MS) compared to those of controls and mild or moderate MS-patients [9]. These results revealed a novel role of ASPRV1 in immune cells and implied its importance in the progression of acute chronic inflammation, especially in experimental autoimmune encephalomyelitis and MS [9]. Although the role of ASPRV1 in epidermal cells is well-studied, future studies need to be addressed to better understand its function and contribution of neutrophils to MS.

While some functional properties have already been revealed [1,2,5], further characterization of ASPRV1 is justified by its potential importance in the development and treatment of some skin [8] and immune disorders, including MS [9,10,11]. Better understanding of protein characteristics may aid studies on protein function, and exploring effects of different protease inhibitors on ASPRV1 may be of potential therapeutic interest. Therefore, this study was done with the aim to express, purify, and characterize both wild-type and mutant SASP28 and SASP14 proteins. In accordance with the previous studies [1,2,5], herein we also expressed the recombinant proteins fused with glutathione S-transferase (GST) tag. In-vitro experiments were performed in order to investigate enzyme kinetic properties, amino acid preferences of S2 and S3 binding sites, dimer stability, and the susceptibility of SASP14 towards protease inhibitors. Homology modeling was used to predict the quaternary structure of SASP14, which was used to interpret results of specificity and dimerization studies and correlate the results of in-vitro and in-silico analyses.

## 2. Materials and Methods

### 2.1. Modeling

Secondary structure prediction was performed based on the sequence of SASP14 (UniProtKB: Q53RT3) using PredictProtein [12], JPred4 [13], DSC [14], SOPMA [15], and GOR4 [16] web servers. Hydropathy index values were obtained from the literature [17]. SWISS-MODEL Workspace was applied for template search [18], Modeller 9v13 [19] for homology modeling, while ProSA (Protein Structure Analysis) web server for model evaluation [20]. Crystal structures of equine infectious anemia virus (EIAV) protease (PDBID: 1FMB) [21], and human (PDBID: 3S8I) [22] and yeast (PDBID: 2I1A) Ddi1 proteins [23] were used as templates. The volumes of substrate-binding cavities were calculated based on a previously described method using the SiteID module of Sybyl [24,25]. Calculations were performed using the Sybyl program package (Tripos Inc., St. Louis, MO, USA) run on Silicon Graphics Fuel workstations (Silicon Graphics International, Fremont, CA, USA). Molecular visualizations were made by PyMol Molecular Graphics System (Version 1.3 Schrödinger, LLC, New York, NY, USA). Stability analysis was performed using I-Mutant 2.0 web server [26]. Multiple structure alignment was performed by using mTM-Align web server [27].

### 2.2. Cloning and Mutagenesis

SASP37, SASP28, and SASP14 sequences (GeneID: 151516) were amplified from pCMV6-XL4-asprv1 vector (OriGene, Rockville, MD, USA) by PCR using Phusion high-fidelity polymerase (New England Biolabs, Ipswich, MA, USA) and oligonucleotides (Sigma-Aldrich, St. Louis, MO, USA) summarized in Table S1A. Primer sequences are also available in the public oligonucleotide database of Laboratory of Retroviral Biochemistry (http://lrb.med.unideb.hu/research/oligos). The amplified inserts were purified from agarose gel using QIAquick Spin Kit (Qiagen, Hilden, Germany). After digestion with BamHI and EcoRI restriction endonucleases (New England Biolabs, Ipswich, MA, USA), the inserts were subcloned into pGEX-4T-3 expression vector (GE Healthcare, Chicago, IL, USA) using Quick Ligation Kit (New England Biolabs, Ipswich, MA, USA). The expression vectors were transformed into DH5α competent cells (New England Biolabs, Ipswich, MA, USA), the subclones were then checked by colony PCR using Phusion high-fidelity polymerase (New England Biolabs, Ipswich, MA, USA) and were sequenced using BigDye Terminator (Applied Biosystems, Foster City, CA, USA). SASP28-A189K/N190I and SASP28-A167G/L168G/A189K/N190I autoprocessing site mutants were generated by QuickChange Lightning Multi Site-Directed Mutagenesis Kit (Agilent Technologies, Santa Clara, CA, USA) using oligonucleotides summarized in Supplementary Materials Table S1B. Success of mutagenesis was proved by sequencing in all cases.

### 2.3. Expression and Purification

The expression vectors were transformed into *Escherichia coli* BL21(DE3) competent cells (New England Biolabs, Ipswich, MA, USA) by heat-shock. Expressions of GST-fusion proteins were induced by the addition of 1 mM isopropyl β-d-1-thiogalactopyranoside (IPTG). Cells were harvested by centrifugation at 4 °C for 20 min at 5000 *g* (Sorvall Lynx 4000, Thermo Fisher Scientific, Waltham, MA, USA). Pellets were lysed in lysis buffer (0.01 M Na_2_HPO_4_, 0.15 M NaCl, 0.005 M EDTA, 2% sarcosyl, pH 7.4) followed by sonication (Branson Sonifier 450). Lysates were centrifuged at 10,000 *g* for 20 min at 4 °C (Sorvall Lynx 4000, Thermo Fisher Scientific, Waltham, MA, USA).

Solubilized recombinant GST-fusion proteins were purified in the presence of detergents (0.3% sarcosyl and 3% Triton X-100) by affinity chromatography using Bio-Scale Mini Profinity GST Cartridge (BioRad, Hercules, CA, USA) using an ÄKTAprime (Amersham Pharmacia Biotech, Little Challfont, UK) instrument. Fractions were dialyzed using 6–8 kDa standard regenerated cellulose membrane against PIPES buffer (0.02 M PIPES, 0.001 M EDTA, 0.1 M NaCl, 10% glycerol, 0.5% NP-40, pH 7.0). Protein concentrations were determined by Pierce BCA Protein Assay Kit (Thermo Fisher Scientific, Waltham, MA, USA).

For denaturing SDS-PAGE, samples were complemented with Laemmli sample buffer (containing SDS and β-mercaptoethanol) and then incubated at 95 °C for 7 min. PageBlue Protein Staining solution (Thermo Fisher Scientific, Waltham, MA, USA) was used for gel staining. Densitometry was performed using the freely available GelAnalyzer software (http://www.gelanalyzer.com).

### 2.4. Synthetic Oligopeptides

Oligopeptide substrates representing the naturally occurring matrix/capsid (MA/CA) cleavage site of HIV-1 (VSQNY↓PIVQ) and its P2- and P3-modified variants were in-house-stocks [24,25]. Oligopeptide substrates representing the wild-type and modified pro-FLG linker sequences (see later in Table 1) were ordered from BioBasic. All peptides were dissolved in distilled water, except the P4-phosphorylated GSFLY↓QVSTH peptide, which was dissolved in 50% DMSO.

### 2.5. Protease Activity Assay

The activity assays were initiated by mixing 5 μL (25–600 nM determined based on protein amount) of purified enzyme with 10 μL of incubation buffer (0.25 M Na-phosphate, 2 M NaCl, 5% glycerol, 5 mM dithiothreitol (DTT), pH 5.6), and 5 μL of substrate (0.24–1.2 mM final concentration). The substrate concentration range was selected for kinetic measurements depending on the approximate *K*_m_ values. The reaction mixtures were incubated at 37 °C for 1 h and stopped by the addition of 180 μL of 1% trifluoroacetic acid (TFA). Substrates and cleavage products were separated using Nova-Pak C18 reversed-phase chromatography column (3.9 × 150 mm, Waters, Milford, MA, USA) on Merck Hitachi HPLC system by an HPLC-based assay, using automatic injector. Increasing water/acetonitrile gradient (0–100%) was used for separation in the presence of 0.05% TFA. Kinetic parameters were determined at less than 20% substrate hydrolysis, and data were evaluated using GraphPad Prism 5.01 program (for Windows, GraphPad Software, La Jolla, CA, USA; www.graphpad.com).

### 2.6. Determination of pH and Ionic Strength Optima

To investigate dependence of GST-SASP14-wt activity on pH and ionic strength, cleavage reactions were performed in META buffer (0.05 M 2-(*N*-morpholino)ethanesulfonic acid (MES), 0.1 M Tris-HCl, 0.05 M Na-acetate) using VSQLY↓PIVQ peptide representing P2-Leu variant of HIV-1 MA/CA cleavage site as substrate (190 μM final concentration). For the determination of optimal pH, the buffer contained 2 M NaCl, and pH ranged from 5.0 to 9.0. To study effects of ionic strength on protease activity, NaCl concentration ranged from 0 to 2 M (at pH 6.0).

### 2.7. Determination of Urea Dissociation Constant (UC50)

VSQLY↓PIVQ oligopeptide substrate (1.2 mM final concentration) was used to determine urea dissociation constant in META buffer (0.05 M MES, 0.1 M Tris-HCl, 0.05 M Na-acetate, 2 M NaCl, pH 5.0). META buffer contained urea in final concentration ranging from 0 to 2 M. Reactions were initiated by the addition of GST-SASP14-wt (150 nM).

### 2.8. Inhibition of GST-SASP14 by HIV-1 Protease Inhibitors

Pepstatin A, acetyl-pepstatin [28], indinavir, tipranavir, saquinavir, nelfinavir, darunavir, lopinavir, and amprenavir [29] were in-house stocks. Acetyl-pepstatin was dissolved in acetic acid (50%), while all other inhibitors in dimethyl sulfoxide (DMSO). Control reactions contained no inhibitor, only the solvent of the inhibitor was added to the reaction mixture. Substrate conversion measured for control was considered to be 100%. Inhibitors were diluted using phosphate buffer (0.25 M Na-phosphate, 2 M NaCl, 5% glycerol, 5 mM DTT, pH 5.6). VSQLY↓PIVQ oligopeptide was used as substrate (0.46 mM final concentration) for activity measurement of GST-SASP14-wt (150 nM). For screening of inhibitors, final concentration of DMSO was 1%, and the inhibitors were applied in 10 μM final concentration; otherwise, it is indicated. To determine inhibitory constant and the concentration of the active enzyme in the fractions of GST-SASP14 used for kinetic measurements, indinavir was applied in 0–10 μM range of final concentration. Statistical significances were calculated by using GraphPad QuickCalcs *t* test calculator (https://www.graphpad.com/quickcalcs/ttest1.cfm).

### 2.9. Autoactivation

To investigate the autoactivation of ASPRV1, GST-SASP28 enzyme was diluted with reaction buffer (0.25 M Na-phosphate, 2 M NaCl, 5% glycerol, 5 mM DTT, pH 5.6), and two parallel samples were incubated for 0, 5, 15, 30, and 60 min at 37 °C. Following the pre-incubation, one of samples was analysed by SDS-PAGE to determine the ratio of processed and unprocessed forms, while the other was supplemented with VSQLY↓PIVQ oligopeptide substrate (0.48 mM final concentration), and incubated for 37 °C for 5 min. The substrate and cleavage product were separated by an HPLC-based method described above.

### 2.10. Cleavage Site Identification by HPLC-(+)ESI-TOF

For identification of cleavage position in synthetic oligopeptide substrates, reaction mixtures were prepared as described in Section 2.5. The cleavage reactions were incubated at 37 °C overnight, then were analysed by high-performance liquid chromatography coupled to electrospray ionization time-of-flight mass spectrometry (HPLC-ESI-TOF).

The HPLC-MS measurements were carried out by Waters 2695 Separation Module with a thermostable autosampler (5 °C) and a column module (35 °C) (each from Waters, Milford, MA, USA). A VDSphere PUR 100 C18-M-SE column (4.6 × 150 mm, 5 μm) was applied with gradient elution. Mobile phases A and B were water (with 0.02% formic acid) and acetonitrile (with 0.02% formic acid), respectively, with a flow rate of 1.5 mL/min. Two methods were used, the gradient conditions of method 1 were set as follows: initially, 100% of A, 0.0–13.3 min, 20% A and 80% B, 13.3–18.5 min 30% A and 70% B, 18.5–21.0 min 50% A and 50% B, 21.0–25.0 min 100% B. The gradient conditions for method 2 were set as follows: initially 100% of A and it was held for 10 min, 10.0–25.0 min 7% A and 93% B, 25.0–30.0 min12% A and 88% B, 30.0–34.0 min 50% A and 50% B, 35.0–45.0 min 100% B. Both method 1 and method 2 applied linear change in gradient profiles. 10 μL was injected from each sample.

A MicroTOF-Q type Qq-TOF MS instrument (Bruker Daltonik GmbH, Bremen, Germany) equipped with ESI ion source was used in positive ion mode. The spray voltage was 4 kV, while nitrogen was used as drying (200 °C) and nebulizing gas (1.6 bar). All measurements were recorded by means of a digitizer at a sampling rate of 2 GHz. The mass spectra were calibrated externally using the ESI-tune mix from Bruker.

### 2.11. Cleavage Site Identification by MALDI-TOF-MS

In order to identify cleavage position in GST-SASP28 recombinant protein, we incubated the double mutant and wild type GST-SASP28 and GST-SASP14 proteins, respectively, using the method described in Section 2.9. The purified GST-SASP28 proteins were incubated in reaction buffer overnight at 37 °C. After the incubation, the samples were dialyzed against distilled water and then concentrated using Eppendorf Concentrator plus system. Sample volumes were reduced from 5 mL to 300 μL. To further concentrate and desalt the samples for matrix-assisted laser desorption/ionization time-of-flight mass spectrometry (MALDI-TOF-MS) measurements, we used C18 ZipTip pipette tips (ZTC18S096, Sigma-Aldrich, St. Louis, MO, USA).

The MALDI-TOF MS measurements were carried out by a Bruker Autoflex Speed mass spectrometer. Linear mode was used for all samples, where the ion source voltage 1 and ion source voltage 2 were 19.5 kV and 18.3 kV, respectively. The instrument was equipped by a solid phase laser (355 nm, ≥100 μJ/pulse) which was applied at 500 Hz and 5000 shots were summed. Spectra were calibrated by Protein Calibration Standard I. from Bruker.

The samples were prepared with 2,5-dihydroxybenzoic acid (DHB) matrix. The matrix was dissolved in 50% aqueous acetonitrile supplemented with 0.1% TFA (100 mg/mL). 1 μL of matrix and the same amount of the sample were deposited onto the plate and were allowed to dry.

## 3. Results

### 3.1. Secondary Structure Prediction

Based on the results of secondary structure predictions, SASP14 shares its overall secondary structural arrangement with the homodimeric aspartyl proteases (Figure 1). SASP14 was predicted to contain an additional helical insert, similarly to equine infectious anemia virus (EIAV) [21] and DNA-damage-inducible 1 (Ddi1) proteases [23]. This short helix is located in the proximity of the consensus catalytic sequence motif and cannot be observed in most retroviral aspartic proteases [30]. Prediction implied that the homodimeric SASP14 has a six-stranded dimer interface, because the C-terminal region was predicted to contain three β-strands connected by short loops (Figure 1). Results of secondary structure predictions performed by different methods were in agreement with each other and implied the presence of three C-terminal β-strands (Figure S1).

**Figure 2:**
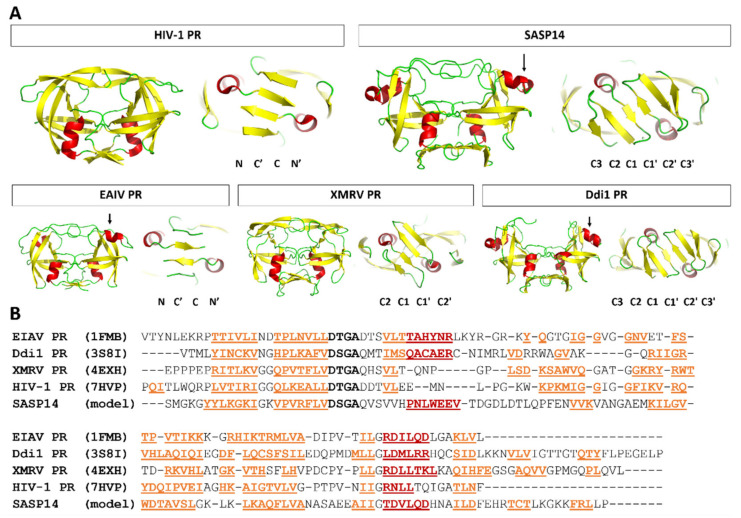
Overall fold of SASP14 resembles to those of homodimeric aspartic proteases. (**A**) Structures are presented based on the crystal structures of HIV-1 (PDBID: 7HVP), EIAV (PDBID: 1FMB), XMRV (PDBID: 4EXH), and human Ddi1 (PDBID: 3S8I) proteases, whereas homology model is shown for human SASP14 homodimer (1–124 residues). Front views of the proteases and enlarged dimer interfaces are shown, organizations of N- and C-terminal β-sheets in dimer interfaces are also indicated. Arrows indicate the additional helical inserts in EIAV, Ddi1, and SASP14 proteases. Color codes: yellow, β-strand; red, α-helix; green, loop. (**B**) Structure-based alignment of protease sequences. Secondary structural organizations are shown based on crystal structures, and based on prediction for SASP14. Color codes: yellow, β-strand; red, α-helix; active site motif residues, black and bold.

SASP14 contains a D-S-G-A sequence that corresponds to the conserved D-S/T-G-A active-site motif of retroviral proteases. Similarly, to human Ddi1 PR, SASP14 also contains a Ser residue in the consensus active site motif (Figure 2B). A Ser residue in this position can provide looser “fireman’s grip” interactions as compared to a Thr residue, which is characteristic for most retroviral proteases [30]. In agreement with this, the sequence motif of the consensus helix in SASP14 is also more similar to those of Ddi proteases. Most retroviral proteases contain a G-R-N/D motif in the consensus helix, the Arg residue is highly conserved in this position and can form H-bond interactions with the other subunit, which contributes to dimer stabilization. Retroviral-like Ddi1 and Ddi2 PRs were found to lack a charged residue in the corresponding position [30], and SASP14 was also found to have a similar triad that contains no Arg (Figure 2B).

### 3.2. Homology Modeling

The three-dimensional structure of SASP14 has not been solved experimentally to date; therefore, a putative 3D structure for the homodimeric enzyme was predicted by homology modeling (Figure 2A). Although a homology model of SASP14 has already been published previously, the EIAV PR structure was used as a single template for prediction and the proposed model structure was not further analyzed [1]. In this study, we used multiple templates to model the quaternary structure of SASP14 and identify residue positions within the homodimeric protease.

The predicted presence of three β-strands at the C-terminus of SASP14 suggested similarity of the dimer interface of the homodimeric SASP14 with that of Ddi1 proteins (Figure 2); thus, Ddi1 proteins were used to build a six-stranded dimer interface for SASP14. The crystal structure of EIAV PR was also used to model the closed conformational flap regions of SASP14, because the flaps of are disordered in the electron density maps of the Ddi1 proteins that were applied as templates [22,23], and does not cover the active site in other available Ddi1 or Ddi2 structures (Figure S2). Furthermore, EIAV PR also contains an additional helical insert (Figure 2), and EIAV and SASP14 proteases share high sequence identity of the residues surrounding the active site [1].

Although the selected templates showed only low sequence identity with SASP14 (<20%), there is a high structural similarity between these proteases [23,30]. In agreement with this, we found that the available Ddi1 and Ddi2 structures are highly similar and differ mainly in flap conformations (Figure S2). It is important to note that possible uncertainties of the models need to be considered during interpretation of the data, as the highest sequence identities between SASP14 and Ddi1/Ddi2 proteases are also close to a sub-optimal template selection (Figure S2).

### 3.3. Mutation Design

Met2 residue (see Figure 1B for SASP14 numbering) was mutated to Ile in all studied SASP14 enzymes to increase protein stability by preventing oxidation of this sidechain. Sequence-based analysis (I-Mutant 2.0) implied slightly stabilizing nature of this mutation, this residue does not constitute a part of any substrate-binding site or the dimer interface. Recombinant SASP14 bearing M2I mutation was used in the in vitro experiments and was considered as wild-type.

In order to investigate whether mutations of self-processing sites may affect autoproteolysis, two cleavage site mutants have been designed for GST-SASP28. The A189K/N190I double mutant contained modified P2 and P1 residues of the N-terminal processing site of SASP14, while the P1 and P1’ residues of the alternative autoproteolytic site was modified in the A167G/L168G/A189K/N190I quadruple mutant (Figure 1). We expected that mutations of the N-terminal SASP14 cleavage site (double mutant) may shift the cleavage position to the alternative site, furthermore, we assumed that the processing at this site can be prevented by the additional mutations (quadruple mutant).

### 3.4. Expression and Purification of GST-Fusion Proteins

**Figure 3:**
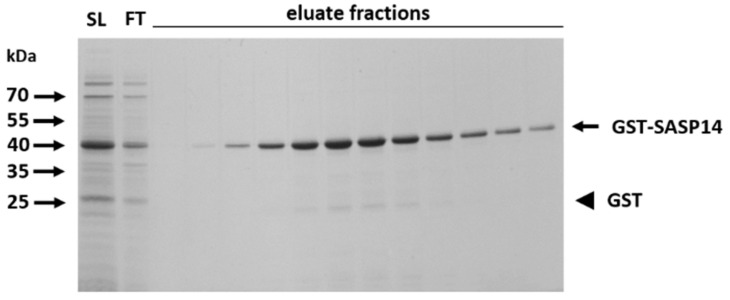
Purification of GST-SASP14 by affinity chromatography. A representative gel shows the SDS-PAGE analysis of soluble lysate (SL), flow-through (FT), and eluate fractions from the affinity-purification of wild-type GST-SASP14. Black arrow indicates GST-SASP14 and GST is shown by black arrowhead.

SASP28 and SASP14 proteins were expressed in a bacterial expression system as recombinant proteins fused to GST tag. We used only GST-SASP28 and GST-SASP14 forms in our experiments because we were unable to purify sufficient amount of SASP37 for characterization, possibly due to the presence of the transmembrane domain. In agreement with this, inefficient expression of SASP37 in *E. coli* cells have already been reported previously [1]. Both GST-SASP28 and GST-SASP14 enzyme forms were solubilized and purified in the presence of detergents, therefore, possible differences between the solubility of the different enzyme forms or mutants were not investigated. During the expression and purification of GST-SASP14 we detected negligible amount of GST in the samples which was not a result of processing (Figure 3) [1], while GST-SASP28 was found to undergo autoproteolysis during its purification (see later in Section 3.11).

### 3.5. Protease Assays by GST-SASP14

Enzyme activity measurements were performed in order to explore biochemical characteristics of ASPRV1, using synthetic oligopeptide substrates representing wild-type or modified versions of HIV-1 protease MA/CA cleavage site (Table 1).

We have tested whether the VSQNY↓PIVQ synthetic oligopeptide—representing the naturally occurring MA/CA cleavage site of HIV-1 protease—is susceptible for the cleavage by GST-SASP14. It was found previously that this sequence can be cleaved efficiently by numerous different retroviral proteases [24,25]. Therefore, we assumed that the wild-type cleavage site may be cleaved efficiently by SASP14, and this peptide can be used for activity measurements.

Primary cleavage reactions showed that VSQNY↓PIVQ peptide is not hydrolyzed by SASP14 if it was incubated for 1 h, however, we observed processing after incubation overnight. A previous comparative specificity study of our laboratory proved that unlike HIV proteases, retroviral proteases usually have hydrophobic S2 pockets; therefore, replacement of P2-Asn in the wild-type HIV-1 MA/CA cleavage site sequence by a hydrophobic residue was found to increase cleavage efficiency of most retroviral proteases [24]. Most remarkable effect of this substitution was observed for human T-cell leukemia virus type 1 (HTLV-1) and human foamy virus (HFV) proteases, which were unable for the cleavage of the unmodified peptide but hydrolyzed the P2-Leu variant. Additionally, P2-Leu mutant was remarkably (~200-fold) better substrate of bovine leukemia virus (BLV) PR than the oligopeptide with the wild-type sequence [24]. Due to this, we expected that a substrate containing a hydrophobic residue in P2 position may be hydrolyzed by GST-SASP14 and tested P2-Leu mutant oligopeptide as substrate.

**Table 1:** Catalytic efficiency of GST-SASP14. Arrows indicate cleavage position in cleavage site sequences. References are indicated for those values that have been published previously.

Cleavage Site	Sequence	Protease	K_M_ (mM)	k_cat_ (s^−1^)	k_cat_/K_M_ (mM^−1^ s^−1^)	Reference
HIV-1 MA/CA wt	VSQNY↓PIVQ	GST-SASP14	not hydrolyzed			
		HIV-1	0.15	6.8	45.3	[31]
		HIV-2	0.18	6.2	34.4	[32]
		HTLV-1	not hydrolyzed			[33]
HIV-1 MA/CA P2-Leu	VSQLY↓PIVQ	GST-SASP14	1.26	0.034	0.027	
		HIV-1	0.12	0.4	3.3	[32]
		HIV-2	0.17	3.4	20.0	[32]
		HTLV-1	0.26	0.05	0.2	[33]

In contrast with the wild-type peptide, the modified peptide containing P2-Leu mutation was hydrolyzed using 1 h incubation (Table 1); therefore, we used this P2-Leu variant in the subsequent measurements to study dependence of protease activity on different conditions. For specificity analysis, other variants have also been tested; the results of in vitro and in silico specificity analysis are discussed later. Catalytic efficiency of GST-SASP14 on VSQLY↓PIVQ peptide was significantly lower than that of HIV-1, HIV-2 and HTLV-1 proteases (Table 1).

### 3.6. Determination of pH and Ionic Strength Optima

While the extracellular pH is neutral in the deeper layers of skin epidermis, both the extracellular and the intracellular pH is more acidic close to the surface of the skin [3,34]. ASPRV1 functions in the *stratum corneum*, which suggested that it has acidic pH optimum. In accordance, previous studies on ASPRV1 implied acidic pH optimum for human [1] and mouse enzymes [2]. Optimal pH for human SASP14 has not been determined previously, therefore, a series of buffers was used for determination of pH optimum.

**Figure 4:**
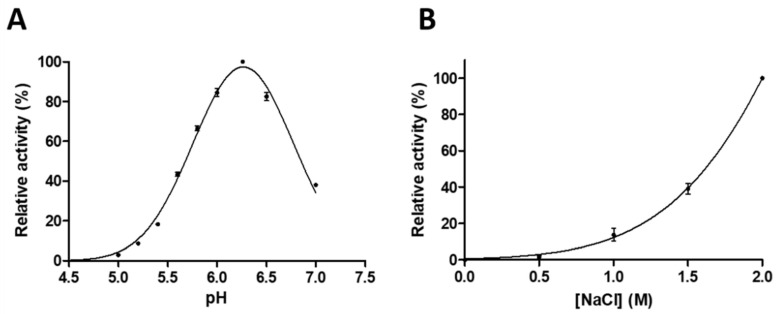
GST-SASP14 catalytic activity depends on pH and ionic strength. Enzyme activity was measured at increasing (**A**) pH (*n* = 2) or (**B**) ionic strength (*n* = 3). We used HPLC detection of substrate cleavage as described in Materials and methods. Values in parentheses represent the number of independent experiments. Error bars represent SD.

We found that the pH optimum of GST-SASP14 (pH_opt_ = 6.27 ± 0.02) was close to the physiological pH of *stratum granulosum* and that the protease may exhibit activity at the weakly acidic pH of *stratum corneum*. This weakly acidic pH optimum (Figure 4A) is similar to that of human foamy virus (HFV) protease (pH 6.6–6.8) [35], but is higher as compared to that of HIV-1 PR (pH 4–6) [36]. SASP14 activity was also found to be boosted by high ionic strength (Figure 4B), similarly to the HIV-1 and HFV proteases [35,36].

### 3.7. Determination of Urea Dissociation Constant (UC_50_) of GST-SASP14

**Figure 5:**
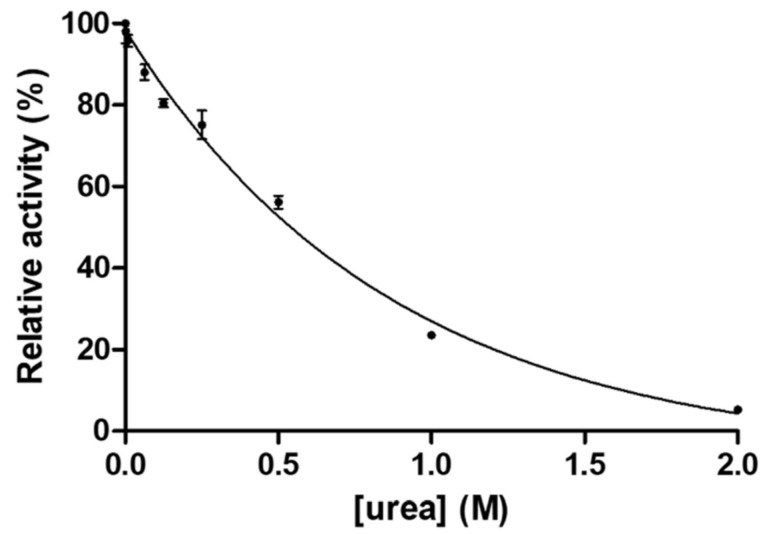
Dependence of GST-SASP14 activity on urea concentration. Enzyme activity was measured at increasing urea concentration. We used HPLC-based detection of substrate cleavage as described in Materials and methods. Error bars represent SD (*n* = 2).

Dimer stability of GST-SASP14 was investigated (Figure 5) by determination of the urea concentration causing 50% loss of enzyme activity (also referred as urea dissociation constant, UC_50_). The obtained stability values were compared with the values reported previously for HIV-1, XMRV [28], and Ty1 retrotransposon proteases [37].

The UC_50_ of GST-SASP14 was found to be lower as compared to HIV-1 PR, but was higher those of Ty1 and XMRV PRs (Table 2).

Differences in dimer stabilities of SASP14, Ty1, HIV-1, and XMRV proteases can be explained in part by the dimer interface organizations of these enzymes. As we found recently, dimer interface organization is main determinant of intermonomeric interactions, and the proteases (e.g., HIV-1 PR) having alternating N- and C-terminal β-strands at the dimer interface show significantly higher contact density as compared to those proteases (e.g., XMRV PR) of which interface comprise only C-terminal β-sheets, without alternation [30]. Dimer interface organization of GST-SASP14 appear to closely resemble that of XMRV PR [38], but XMRV contains a four-stranded dimer interface, while SASP14 has a six-stranded dimer interface organization (Figure 2). None of these dimer interfaces show interdigitation of the C-terminal β-sheets, thus can provide only lower dimer stability as compared to HIV-1 PR (Table 2).

### 3.8. Amino Acid Preferences

**Figure 6:**
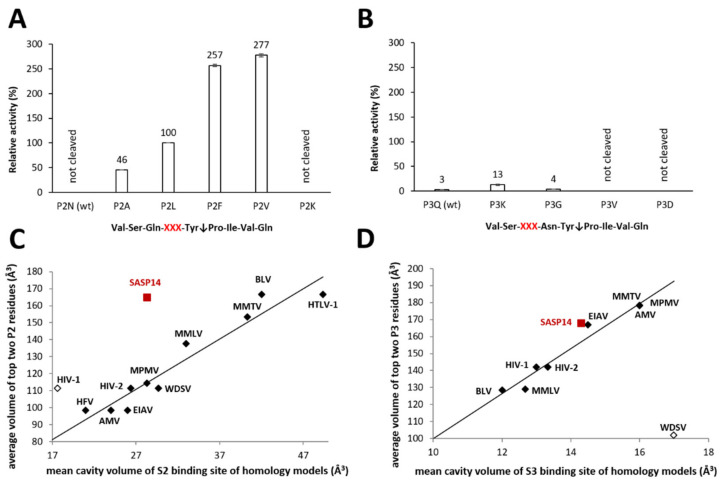
P2- and P3 residue preferences of GST-SASP14. The synthetic oligopeptides used for activity measurements represented wild-type and P2- or P3-modified variants of HIV-1 MA/CA (VSQNY↓PIVQ) cleavage site, modified positions are indicated by XXX. Relative activities were determined for the wild-type substrate and for its P2- (**A**) and P3-modified variants (**B**) by incubating reaction mixtures for 1 and 16 h, respectively. Activity determined for P2-Leu variant was considered to be 100%. Error bars indicate SD (*n* = 2). Figure part (**C**,**D**) was prepared in part based on previously published data [24,25]: the average volumes of two residues for which the measured relative activity was the highest are plotted against the mean cavity volumes of S2 (**C**) and S3 subsites (**D**) of various retroviral proteases. Values determined for SASP14 are shown by red squares, values of previously published dataset are shown by diamonds. The black symbols show values that were used for correlation, while opened diamonds indicate values excluded from the former analysis. For SASP14, average residue volumes of top P2 residues (Phe and Val) of the cleaved substrates are shown (**C**), whereas volume of Lys is plotted against the mean cavity volume of P3 binding site (**D**). Additionally, compositions of the S2 and S3 substrate-binding cavities are shown in Table S2.

To investigate amino acid preferences of S2 and S3 substrate-binding sites of SASP14, we tested a series of synthetic oligopeptide substrates with amino acid substitutions in the P2 and P3 positions, respectively (Figure 6). Modeled enzyme-substrate complexes were also analyzed to elucidate the observed differences. The model quality was assessed by ProSA web server, and the overall model qualities obtained for the homology model and template were similar. The z-score calculated for the model was slightly lower than those obtained for the templates and was within the range of experimentally determined protein structures (Figure S3). To probe the possible role of the ASPRV1 residues in substrate binding, a VSQNY↓PIVQ substrate was modified in silico by the substitution of the P2 and P3 residues, followed by the minimization of the enzyme-substrate complexes, and using the minimization procedure which was applied previously to calculate substrate-binding cavity volumes [24,25].

Our previous comparative studies on retroviral proteases have suggested an important role of the S2 subsite [24]. VSQNY↓PIVQ substrate was not processed during 1 h incubation, while modified versions of this peptide containing hydrophobic residues in P2 position were cleaved efficiently (Figure 6A). No cleavage was observed for the P2-Lys variant, even incubating the reaction mixture for 16 h.

Our results are in agreement with those of a previous study that revealed amino acid preferences of numerous retroviral proteases [24]. Here we found that SASP14 also has a hydrophobic S2 pocket (Table S2), which is larger as compared to that of HIV-1 (Figure 6C). Cleavage reactions also showed preferential cleavage of substrates containing hydrophobic residues in P2 position, while wild-type HIV-1 MA/CA cleavage site was not cleaved if it was incubated only for 1 h. Compared to HIV-1 PR, the P2 and P2′ residues of the ASPRV1 natural cleavage site sequences are more hydrophobic, while volumes of substrates residues are highly similar in these positions (Figure S4). Specificity matrix available in MEROPS database [39] also shows preference for hydrophobic P2 and P2′ residues (https://www.ebi.ac.uk/merops/cgi-bin/pepsum?id=A28.004). Based on prediction, SASP14 shows no strong correlation of P2 residue- and S2 cavity-volumes (Figure 6C), which discrepancy may be possibly caused by the uncertainty of the model.

The turnover of the tested P3-modified variants was hardly detectable after 1 h incubation; therefore, the reaction mixtures were incubated for 16 h. P3-Val and -Asp mutants were not cleaved after overnight incubation, and conversion of the wild-type and P3-Gly variant substrates were also only negligible. Out of the tested P3-variants, P3-Lys mutant was cleaved most efficiently, however, the substrate conversion was significantly lower than the turnover observed for P2-Leu mutant after 1 h incubation (Figure 6B). This implies only a low preference for a P3-Lys substituted peptide. In accordance with the predicted larger volume of S3 binding site (Figure 6D) and the predicted interactions of a Lys residue with the enzyme (Figure S5), binding of a Lys residue to the S3 site was predicted to be possibly favorable in the case of the VSKNY↓PIVQ substrate. In silico analyses implied possible involvement of both N- and C-terminal protease residues in substrate binding. In the model structure, negatively charged glutamate residues located in the C-terminal region (^130^EDEFDL^135^) are in close proximity to the P3-Lys residue, enabling the formation of hydrogen bonds with a large and positively charged side chain (Figure S5). The proposed composition of the substrate-binding site (Table S2) and hydrophobicity profiles of ASPRV1 PR cleavage site sequences (Figure S4) also indicated preference for non-hydrophobic residues at the S3 site. Future studies are needed to prove our findings by studying amino acid preferences not only in the context of MA/CA cleavage site, but using series of other cleavage site sequences.

**Figure 7:**
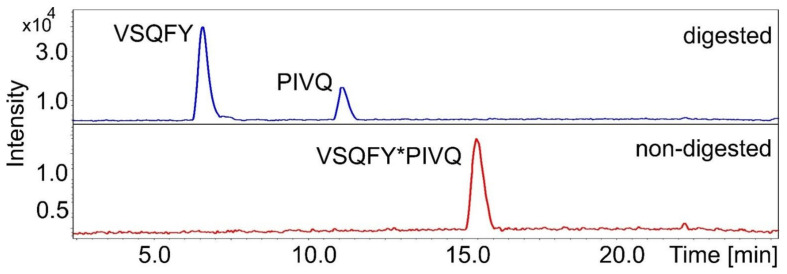
The base-peak chromatograms of digested and non-digested (blank) P2-Phe substrates. Further compounds were not identified. Asterisks indicate cleavage position.

In order to identify the cleavage positions, both the non-digested synthetic oligopeptide substrates and the proteolytic fragments were analyzed by HPLC-ESI-TOF. The retention time and *m*/*z* of the substrates were determined from blank samples, while fragments were identified from digested ones. In every case, the calculated molecular weights were in good agreement with the experimentally determined ones (Table 3). The fragments of P2A peptide eluted together using method 1 parameters, therefore, another chromatographic method (method 2) was used that resulted in different retention times. The analysis proved that both the wild-type and P2- or P3-modified variants are also cleaved between P1-Tyr and P1′-Pro residues, and both SASP14 and HIV-1 PR has the same cleavage position in the HIV-1 MA/CA cleavage sites sequence (Table 3), as was expected. Further fragments were not detected in any chromatogram, indicating that the substrates are not cleaved at alternative positions. As an example, Figure 7 shows the base-peak chromatograms of the P2-Phe samples. Determination of in-vitro cleavage positions confirmed the results of in silico specificity analyses, and is in agreement with the calculations made on the enzyme-substrate complex structures.

To our knowledge, the ability of ASPRV1 for processing of the herein described HIV-1 MA/CA cleavage sites has not previously been published elsewhere. Here, we report that the tested P2 variants containing a hydrophobic residue in P2 position of HIV-1 MA/CA cleavage site can be cleaved efficiently by SASP14, while the wild-type sequence and the tested P3-variants are considered to be inefficient substrates. The increasing number of known cleavage sites may help in better understanding enzyme specificity and may support the identification of potential target proteins. The filaggrin is the only known natural substrate of ASPRV1. While other substrates are still unknown, the identification of additional proteolytic targets may be necessary to understand the function of ASPRV1 in neutrophils [9].

### 3.9. Effect of Filaggrin-Processing Site’s Phosphorylation on Processing by GST-SASP14

Filaggrin has been reported to undergo phosphorylation, and the proposed role of phosphorylation of the FLG units or the linker sequences is to prevent premature processing by making the cleavage sites inaccessible for proteases [40]. Based on data available in PhosphoSite database [41], mainly the conserved P1-Tyr residues are phosphorylated in the linker sequences of human FLG, and phosphorylations of P6, P3′, and P4′ residues are also shown. Phosphorylation of P4-Ser residues have also been reported [42], but the effect of this modification on proteolytic processing was not investigated experimentally to date. The phosphorylation of P1-Tyr residue was found to prevent the hydrolysis of the modified substrate by HIV-1 PR, and molecular modeling studies also revealed that binding of a too large and negatively charged P1-phospho-Tyr residue to the S1 binding site is unfavorable [43].

We assumed that P1-Tyr phosphorylation may have a similar effect in the case of ASPRV1; thus, we tested a P4-phosphorylated version of GSFLY↓QVSTH substrate. This substrate was not soluble in water, and thus was dissolved in dimethyl sulfoxide (50% DMSO). The synthetic oligopeptide substrate representing the wild-type GSFLY↓QVSTH sequence was cleaved efficiently (Figure S6), the cleavage position within this sequence has already been determined previously by N-terminal amino acid sequencing [5]. The P4-Ser phosphorylated variant of the FLG-linker cleavage site was inefficient substrate for GST-SASP14 (Figure S6). In contrast with this, phosphorylation of P4-Ser residue has been reported to have only moderate effect in the case of HIV-1 PR [43]. The P4-Ser residue is highly conserved in pro-FLG linker cleavage site sequences [42], which implies that the phosphorylation in this position may possibly prevent the unwanted cleavage and contributes to the regulation of pro-FLG processing.

### 3.10. Inhibition of SASP14 by HIV-1 Protease Inhibitors

It was reported previously that in the case of antiretroviral therapies the unwanted inhibition of ASPRV1 may be responsible for the cutaneous side effects of indinavir treatment, because ASPRV1 shares the general fold with retroviral proteases; therefore, it is potentially susceptible for inhibition by HIV-1 PR inhibitors [1].

To study the effects of inhibitors on purified GST-SASP14, we tested seven inhibitors approved by FDA for use in highly active antiretroviral therapies (HAART): indinavir, tipranavir, saquinavir, nelfinavir, darunavir, lopinavir, and amprenavir. Furthermore, pepstatin A and acetyl-pepstatin being potent inhibitors of aspartyl proteases were also tested.

**Figure 8:**
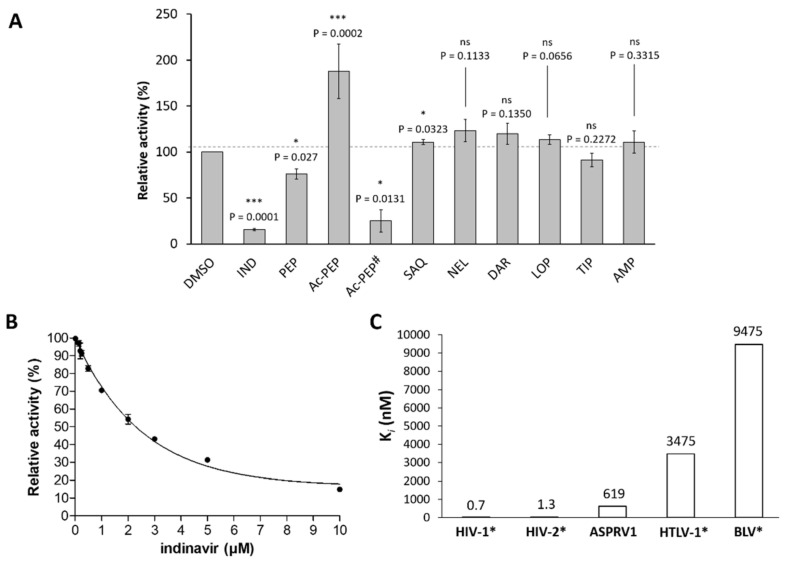
Indinavir is an inhibitor of ASPRV1. (**A**) Relative activity of GST-SASP14 measured in the presence of inhibitors. Enzyme reactions were performed by incubating samples at 37 °C for 1 h using VSQLY↓PIVQ peptide as substrate. Inhibitors: INV, indinavir; PEP, pepstatin A; Ac-PEP, acetyl-pepstatin; SAQ, saquinavir; NEL, nelfinavir; DAR, darunavir; LOP, lopinavir; TIP, tipranavir; and AMP, amprenavir. All inhibitors were applied in 10 μM final concentration. # For Ac-PEP, 15.5 μM final concentration was also applied. Activity of control sample—containing DMSO instead of inhibitor—was considered to be 100%. Symbols indicating significance: ns, *p* > 0.05; *, *p* ≤ 0.05; ***, *p* ≤ 0.001. Error bars represent SD (*n* = 2). (**B**) Activity of GST-SASP14 was measured at increasing indinavir concentration, using HPLC detection of substrate cleavage. Error bars represent SD (*n* = 2). (**C**) *K_i_* value of indinavir determined for ASPRV1 is compared to those of some retroviral proteases. * *K_i_* values for HIV-1 and HIV-2 [46], BLV [47], and HTLV-1 proteases [48] have been reported previously.

Bernard and its coworkers [1] found that the autoactivation of ASPRV1 can be inhibited by indinavir even at 100 μM final concentration. In good agreement with these results we also observed that indinavir can inhibit GST-SASP14, but at significantly lower final concentrations. As indinavir was found to be the most effective against wild-type GST-SASP14 out of the tested inhibitors (Figure 8A), the inhibition constant was determined for this inhibitor (Figure 8B). The *K_i_* was found to be much higher than in the case of HIV-1 and HIV-2 proteases, but significantly lower than inhibitory constant in the case of BLV or HTLV-1 proteases (Figure 8C).

Saquinavir and amprenavir have already been reported to be inefficient inhibitors of ASPRV1 [1], and herein we proved that these inhibitors are unable to inhibit GST-SASP14.

It has been described previously that the classical aspartic protease inhibitor pepstatin A has no effect on self-processing of ASPRV1 even at 1000 μM final concentration [1]. In contrast with this, we found that pepstain A is able to inhibit cleavage of synthetic peptide substrate by GST-SASP14 at significantly lower final concentration (10 μM), but has lower inhibitory potential compared to that of indinavir (Figure 8A).

Inhibitory potentials of tipranavir, lopinavir, darunavir, nelfinavir, and acetyl-pepstatin on ASPRV1 has not been reported to date. Screening of inhibitors showed no effect of tipranavir, lopinavir, darunavir, and nelfinavir on the activity of GST-SASP14 (Figure 8A). Acetyl-pepstatin caused a significant decrease of enzyme activity at 15.5 μM final concentration, but interestingly, the protease activity was boosted if it was applied at lower concentration (10 μM) (Figure 8A). It has been described previously that binding of acetyl-pepstatin to HIV-1 PR can stabilize the dimeric structure [44]. Crystallographic studies showed that acetyl-pepstatin binds exclusively to the active sites of HIV-1 [45] and XMRV proteases [28], but to our knowledge, binding of the inhibitor to other enzyme surfaces has not been described, and elevated proteolytic activity in the presence of this inhibitor was also not reported for any retroviral or retroviral-like proteases.

**Figure 9:**
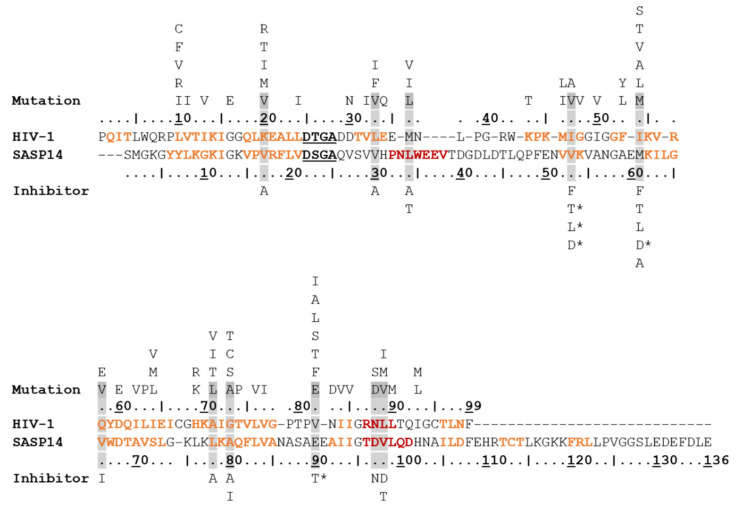
Residues of SASP14 corresponding to resistance mutations of HIV-1 PR. Aligned sequences of HIV-1 and SASP14 proteases are coloured according to secondary structural organization (based on crystal structure and prediction, respectively): β-sheets are orange, and α-helices are red. Alignment with other proteases are shown in Figure 2. The catalytic motif residues are bold and underlined in the sequences. Resistance mutations of HIV-1 PR are shown above the sequence based on Weber et al. [49]. The resistance mutation residues of HIV-1 PR that can be observed at the equivalent positions of SASP14 are highlighted by grey background. Asterisks mark those inhibitors for which the highlighted mutations are considered to be major resistance mutations in HIV-1 PR. Inhibitor names are abbreviated as follows: A-atazanavir, D-darunavir, F-fosamprenavir, I-indinavir, L-lopinavir, N-Nelfinavir, and T-tipranavir.

Similarly to the Ty1 retrotransposon protease of budding yeast *Saccharomyces cerevisiae* [37], SASP14 cannot be inhibited by amprenavir, darunavir, lopinavir, nelfinavir, saquinavir, and tipranavir. We investigated whether resistance mutations of HIV-1 PR may occur in equivalent positions of SASP14 (Figure 9). We found that there are multiple residues in SASP14 that correspond to major or minor resistance mutations of HIV-1 PR. Out of these, only three are binding site-forming residues in HIV-1 PR: I47 in S2 and S4 cavities, Q58 in S4 cavity, while V82 in S1 cavity [24,25]. V66 and A80 residues of SASP14 correspond to Q58V and G73A minor resistance mutations of HIV-1 PR, respectively [49], but despite this SASP14 can be inhibited by indinavir.

### 3.11. Autoprocessing of Different SASP28 Forms

It has already been reported that SASP28 precursor protein undergoes proteolysis, which releases SASP14 [1,2]. Proteins bearing P5 and P4 mutations (I186T and V187I, respectively) of the N-terminal autoprocessing site of SASP14 (IVFAN↓SMGKG) were studied previously by Matsui and its coworkers, who observed elevated self-processing for I186T mutant, while V187I mutant showed lower autoproteolytic activity compared to wild-type GST-SASP28 [5].

**Figure 10:**
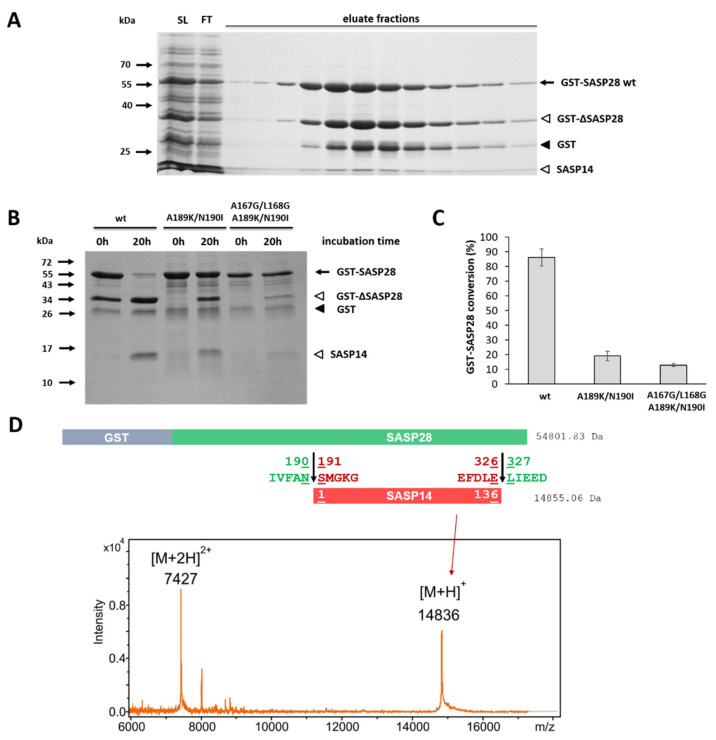
Autoproteolysis of different GST-SASP28 forms. (**A**) A representative gel image shows the SDS-PAGE analysis of soluble lysate (SL), flow-through (FT), and eluate fractions from the affinity-purification of wild-type GST-SASP28. (**B**) All purified GST-SASP28 enzyme forms were incubated for 0 and 20 h. Black arrow indicates full-length GST-SASP28 wt, white arrowheads indicate GST-∆SASP28 and SASP14 as autoproteolytic cleavage fragments, while GST is shown by black arrowhead in the representative gel image. (**C**) After the incubation of GST-SASP28 enzymes (see figure part **B**), densitometry was performed to determine conversion of precursors. The conversion of was determined by comparing the band intensity of the precursor before and after incubation. Band intensity of the non-incubated precursor was considered to be 100% for each enzyme form. Error bars represent SD (*n* = 2). (**D**) Identification of SASP14 released by autoproteolysis of GST-SASP28 precursor. After self-processing, SASP14 was detected by MALDI TOF MS. Calculated molecular weights are shown together with the schematic representation of processing, while MS spectrum shows the identified fragment. Unedited gel images are represented in Figure S7.

We also observed that GST-SASP28 enzymes undergo autoproteolysis during expression and purification, as it is represented in the example of GST-SASP28 in Figure 10A. After autoproteolysis of wild-type GST-SASP28, MALDI-TOF-MS analysis was performed. The good agreement of calculated and measured molecular weights proved that SASP14 is released from the precursor by cleavages at the known N- and C-terminal cleavage sites (Figure 10C).

It has been reported that autoprocessing of ASPRV1 may occur at an alternative site (A167↓L168), as well, after the removal of the N-terminal cleavage site (N190↓S191) [1]. Therefore, in order to investigate the alternative cleavage site we designed GST-SASP28-A189K/N190I and GST-SASP28-A167G/L168G/A189K/N190I mutant. The modifications of processing sites were expected to cause altered processing profile, because the hydrophobicity profiles implied preference for a hydrophobic P2 and a hydrophilic P1 residues for ASPRV1 cleavage sites (Figure S4).

Self-processing of wild-type and mutant GST-SASP28 proteins was studied by incubating the purified proteins at 37 °C in the buffer used for activity measurements (Figure 10B). We found that the introduced mutations failed to prevent self-processing, but the autoproteolysis of mutant GST-SASP28 enzymes was less efficient than that of the wild-type. Based on gel images, the cleavage products showed no altered molecular weights (Figure 10B), which indicated that the cleavage site has not been shifted from 190↓191 to the 167↓168 position upon mutations. In agreement with this, we did not identify such fragments by MALDI-TOF-MS which would be released by cleavage at the alternative cleavage site in GST-SASP28-A189K/N190I double mutant.

As it was expected, mutation of the P2 and P1 residues to hydrophilic (A189K) and hydrophobic (N190I), respectively, caused less efficient self-processing (Figure 10B). Although, we observed impaired self-processing for the mutants compared to the wild-type, the A189K/N190I mutations did not abolish the cleavage in 190↓191 position in repeated experiments (Figure 10B).

A quadruple mutant GST-SASP28 protein containing the mutation of the alternative cleavage site was also designed, and elimination of side chain-mediated interactions at P1 and P1′ sites by A167G and L168G mutations were expected to inhibit proteolysis and prevent cleavage at the alternative site. While we observed no cleavage at the alternative cleavage site in the case of double mutant precursor, it was not possible to study the effects of mutations at this cleavage site in the case of the quadruple mutant.

Self-processing of GST-SASP28 proteins was studied at 37 °C, the extent of processing was estimated by densitometry of gel images (Figure 10C). Almost complete conversion was observed only for the wild-type. Despite modification of autoproteolytic cleavage sites, the mutants still showed autoproteolysis (Figure 10B). While >80% of wild-type GST-SASP28 was processed, we observed >10% self-processing for the mutant proteins even after 1 h incubation. The results implied that simultaneous mutation of P2 and P1 residues (A189K/N190I) failed to impair processing at N190↓S191, and the quadruple mutant also retained its ability self-processing. We assumed that unwanted presence of SASP14 in the mixtures would distort the results of kinetic measurements; therefore, the kinetic parameters for the GST-SASP28 enzymes were not determined. Previous studies on GST-SASP28 showed that V187I mutation caused impaired self-processing of the precursor and decreased its ability for the cleavage of profilaggrin substrate [5]. Due to this correlation, we also assumed that both mutant GST-SASP28 proteins—showing decreased autoproteolytic activity—may cleave the oligopeptide substrates less efficiently.

### 3.12. Autoactivation

Although there were some attempts to prove the autoactivation of ASPRV1 earlier [1], we consider that the previously reported experiments proved only autoproteolysis of ASPRV1 but not autoactivation, because the activities of SASP28 and SASP14 forms have not been compared consequently.

**Figure 11:**
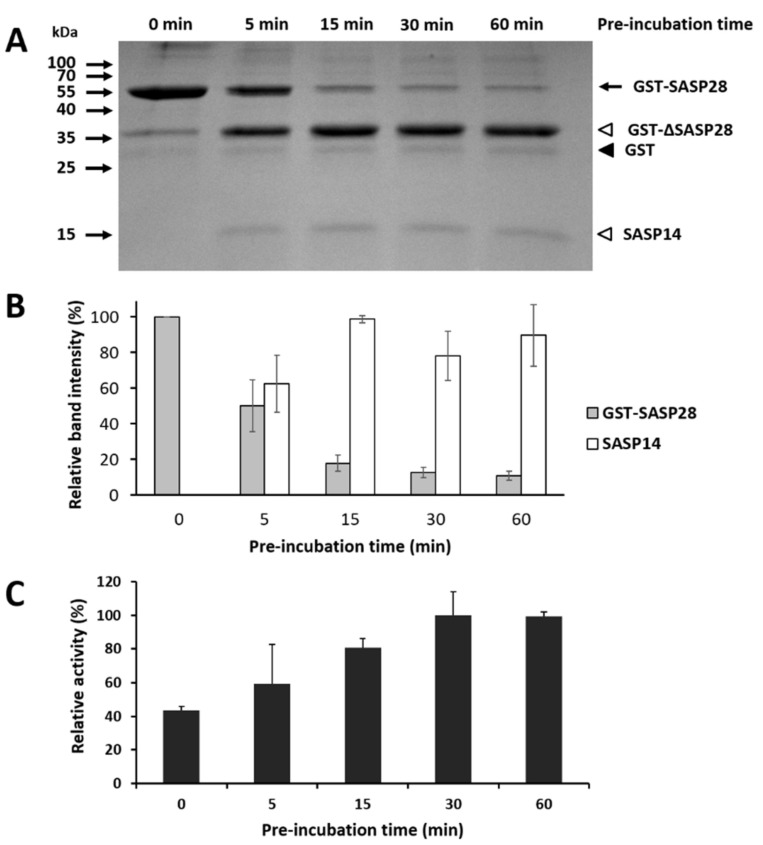
ASPRV1 is activated by autoproteolysis of the precursor. (**A**) A representative SDS-PAGE gel image shows self-processing of the full-length GST-SASP28 precursor and the release of SASP14. The purified GST-SASP28 was pre-incubated in reaction buffer for 0, 5, 15, 30, and 60 min. Black arrow indicates full-length GST-SASP28 precursor, white arrowheads indicate GST-∆SASP28 and SASP14 as autoproteolytic cleavage fragments, while GST is shown by black arrowhead. (**B**) The relative band intensities were determined via densitometry of the gels. For GST-SASP28 and SASP14, the most intense band was considered to have 100% intensity in the case of each gel. Error bars represent SD (*n* = 3). (**C**) The effect of self-processing on enzyme activity was investigated by measuring the hydrolysis of VSQLY↓PIVQ oligopeptide substrate, using an HPLC-based method. Relative activities are plotted as a function of time of pre-incubation for all samples. Error bars represent SD (*n* = 2).

Therefore, in our experiments we investigated ASPRV1 autoactivation in samples containing SASP28 and SASP14 in different ratios. To ensure self-processing and release of SASP14, the GST-SASP28 precursor was pre-incubated up to 60 min at 37 °C, and processing of the precursor was monitored by SDS-PAGE. We found that the GST-SASP28 precursor was the most prevalent enzyme form prior to the incubation, while the processed SASP14 was absent from the sample (Figure 11A). Based on band intensities, during incubation the amount of GST-SASP28 precursor decreased, while SASP14 was released by autoproteolysis (Figure 11B).

After pre-incubation, all samples were complemented with the oligopeptide substrate, and after incubation the relative activities were determined based on substrate turnover. We observed increased substrate conversion as a function of pre-incubation time, and we detected higher enzyme activities for all pre-incubated samples as compared to the non-incubated one (Figure 11C). This implied strong correlation between the amount of SASP14 and enzyme activity.

Boyden et al. found that K199E, R311P, and P314T mutations (K9E, R121, and P124 according to SASP14 numbering, respectively) near the autoproteolytic cleavage sites prevent proper processing and release of SASP14, thus leading to impaired cleavage of filaggrin substrate [8]. This is in agreement with our results and imply significantly lower activity of the improperly- or non-processed precursor as compared to SASP14. Based on this we can conclude that SASP14 has higher activity as compared to GST-SASP28, the autoproteolysis of the precursor causes autoactivation of the protease, and this activation is necessary for efficient cleavage of the substrate.

**Figure 12:**
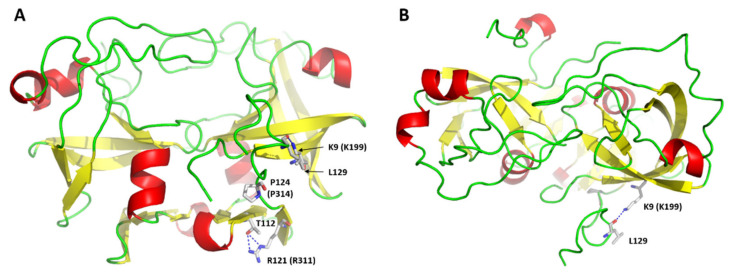
Missense alterations of ASPRV1. Homology model of SASP14 is represented in front (**A**) and top views (**B**). The residues shown by sticks are labeled according to SASP14 numbering, but for the K199E, R311P, and P314T variants [8] the SASP37 numbering is also shown in parentheses. Putative H-bonds are shown by blue dotted lines.

Based on our model, K199 residue is located in the proximity of the N-terminal cleavage site of SASP14, while R311 and P314 residues are close to the C-terminal of the protease domain. These residues are clustered in the protein structure [8], while K199 and R311 residues constitute a part of the first N- and last C-terminal β-strands, respectively, the P314 residues is located in the close proximity of the outer dimer interface strand (Figure 12A).

We assume that impaired abilities of variants for self-processing [8] may be caused by structural changes rather than by the mutations of autoproteolytic cleavage sites sequences, because K199, R311, and P314 residues are located in P9′, P16, and P13 positions, respectively. Thus, point mutations at these positions are unlikely to prevent productive binding of the substrate by the introduction of unpreferred residues relatively distant from the cleavage site. We observed the proximity of N- and C-termini in the modeled enzyme-substrate complex while investigating interactions of P3 residue with the enzyme (Figure S5), and found that K9 residue is also located close to the C-terminal extended region and may interact with a C-terminal residue (Figure 12B). R121 residue was predicted to form H-bond making interaction between the 2nd and 3rd β-strands of the dimer interface (Figure 12A). Both literature data [8] and the proposed model imply that K199, R311, and P314 residues may contribute to conformational integrity of the ASPRV1. Additionally, the mutation-induced changes that affect proteolytic activity highlight the potential importance of the terminal extensions which are characteristic for retroviral-like but not for most retroviral proteases [30]. Experimental approaches need to be used to determine the roles of these residues and reveal how mutations influence structural characteristics of the N- and C-termini, the dimer interface, or the overall domain.

## 4. Discussion

While some functional characteristics have already been investigated previously [1,2,5], the detailed biochemical characterization of ASPRV1 protein has not been performed to date. Therefore, to gain better insight to its properties, in this study we aimed to investigate enzymatic and structural features of this retroviral-like human protease.

Homology modeling revealed that SASP14 shares its overall fold with the retroviral proteases (Figure 2A), and resembles the main structural features of Ddi1 and Ddi2 proteases (Figure 2B), including (i) the presence of an additional helical insert in the proximity of the active site motif, (ii) the six-stranded dimer interface that consists of only C-terminal β-strands, (iii) the active site motif, and (iv) the sequence motif of the consensus helix. The overall structural characteristics imply higher similarity with retroviral-like proteases rather with retroviral proteases, e.g., HIV-1 PR. In agreement with this, the above mentioned features highly resemble those of predicted for the *Saccharomyces cerevisiae* retrotransposon Ty1 protease [37] and for the human retrotransposon-derived paternally expressed gene 10 (PEG10) retroviral-like protease [50], which implies similar structural characteristics for eukaryotic retroviral-like proteases.

In accordance with previous studies [1,5], and in order to ensure comparability of the results, we also studied such wild-type and mutant SASP28 and SASP14 enzymes that were fused with GST affinity tag, which tag enabled affinity purification of the recombinant proteins, as well.

Catalytic efficiency of GST-SASP14 was found to be significantly lower than that of HIV-1, HIV-2 and HTLV-1 proteases (Table 1), and highest activity was measured at slightly acidic pH which was found to be increased with ionic strength (Figure 4). The obtained UC_50_ value implied relatively lower dimer stability of SASP14 as compared to HIV-1 PR (Table 2), the organization of dimer interface was supposed to be a main determinant of this difference.

The amino acid preferences of S2 and S3 substrate-binding sites were studied in silico by analyzing compositions, volumes, and interactions of the binding site cavities, and by comparing hydrophobicity profiles of cleavage site sequence, whereas for in vitro characterization series synthetic oligopeptide substrates were used in cleavage reactions (Figure 6). The structural characteristics were in agreement with the results of protease assays, and revealed the preference of S2 subsite for a hydrophobic residue in SASP14, which is similar to the amino acid preferences of retroviral proteases for the P2 position. Investigation of S3 specificity implied preference for non-hydrophobic residues, and suggested that those N- and/or C-terminal residues which do not constitute a part of dimer interface may be involved in substrate binding.

Testing various protease inhibitors showed that, with the exception of indinavir, SASP14 is not sensitive towards FDA-approved protease inhibitors we tested (tipranavir, saquinavir, nelfinavir, darunavir, lopinavir, and amprenavir) (Figure 8). The inhibitory potential of pepstatin A and acetyl-pepstatin has not been reported for ASPRV1, but based on our findings they are less efficient inhibitors as compared to indinavir. Interestingly, SASP14 activity was inhibited by acetyl-pepstatin at relatively higher concentration (15.5 μM), but at lower concentration it increased enzyme activity (10 μM). Based on literature data [44,45] we assume that acetyl-pepstatin may stabilize dimeric structure, but the detailed investigation of this phenomenon was out of the scope of this study.

Similarly to the retrotransposon Ty1 protease [37] and the human PEG10 protease [50], SASP14 was also found to have a natural resistance against multiple clinical protease inhibitors. Although, we compared the sequence of SASP14 to the sequences of those HIV-1 PR variants which are resistant against various protease inhibitors (Figure 9), the resistance development cannot be interpreted exclusively at the level of resistance residues in equivalent positions, because the similarities and differences of the whole sequences (including active site composition) and structures need also to be considered. For example, we found that saquinavir cannot inhibit SASP14, however, the sequence variations that contribute to the development of resistance in HIV-1 PR against saquinavir are not present in SASP14. Inhibition studies on ASPRV1 may help to understand its contribution to skin barrier dysfunction [1,51], the herein described sensitivity of the enzyme to protease inhibitors reinforces the interest in studying how ASPRV1 could be potentially targeted in the treatment of immune disorders, including MS.

Our results proved that the GST-SASP28 precursor undergoes autoproteolysis (Figure 10), but did not provide evidence for the shift of cleavage to the alternative cleavage site in the enzymes containing mutation(s) of autolytic sites. While the molecular weights of the autoproteolytic fragments showed no differences in the case of the double and quadruple mutant proteins (Figure 10B), in these experiments we failed to identify cleavage position at the alternative processing site which has been reported previously by Bernard et al. [1]. Therefore, the existence of the alternative cleavage site need to be proved by future studies, and the function of cleavage at this site remain to be elucidated. We have investigated the effect of the self-cleavage on enzyme activity, as well. We found that the processed SASP14 has higher activity as compared to the precursor (Figure 11). To the best of our knowledge, we first provide evidence that ASPRV1 undergoes autoactivation due to self-processing of the precursor.

In conclusion, we herein describe a structural and enzymatic characterization of ASPRV1. We hope that our results provide valuable information for a more detailed mapping of differences and similarities between retroviral and retroviral-like proteases. Furthermore, our results can support functional studies of ASPRV1, including studies on clinically relevant variants, and the revealed specificities may support the identification of additional natural substrates. Sensitivity of ASPRV1 towards protease inhibitors may also contribute to the better understanding of enzyme regulation in the treatment of skin disorders and chronic inflammatory autoimmune diseases such as MS and encephalomyelitis.

## Figures and Tables

**Table 2:** Dimer stabilities of GST-SASP14 and retroviral proteases. References are shown for the values that have been published previously.

Enzyme	UC_50_ (M)	Reference
GST-SASP14 wt	0.54 ± 0.06	
HIV-1 PR	1.47	[28]
XMRV PR	0.2	[28]
Ty1 PR	0.05	[37]

**Table 3:** Cleavage site identification in synthetic oligopeptide substrates by HPLC-MS. Oligopeptide substrates—representing wild-type and P2- or P3-modified variants of HIV-1 MA/CA cleavage site—were cleaved by GST-SASP14 PR, by incubating the cleavage reactions at 37 °C overnight. The table shows the *m*/*z* values [M + H]^+^ determined by HPLC-ESI-TOF, the calculated values are shown in parentheses. The non-digested substrates were used as blanks, while the fragments were detected only in the digested samples. *am* and *ac* denote amide- and acid-terminated peptides, respectively. *a* denotes digested peptide measured by method 2 (see details in Section 2.10).

Name	Sequence	Substrate		Fragment 1		Fragment 2	
		*m*/*z* [M + H]^+^ Measured (Calculated)	Rt (Min)	*m*/*z* [M + H]^+^ Measured (Calculated)	Rt (Min)	*m*/*z* [M + H]^+^ Measured (Calculated)	Rt (Min)
**wt *^am^***	VSQNY*PIVQ	1046.567 (1046.563)	11.1	610.283 (610.283)	6.3	455.301 (455.298)	6.7
**P2-Ala *^am^***	VSQAY*PIVQ	1003.565 (1003.557)	11.6	567.282 (567.277)	20.5 *^a^*	455.303 (455.298)	19.9 *^a^*
**P2-Phe *^am^***	VSQFY*PIVQ	1079.594 (1079.588)	15.4	643.306 (643.309)	11.1	455.296 (455.298)	6.5
**P2-Leu *^ac^***	VSQLY*PIVQ	1046.584 (1046.588)	15.6	609.324 (609.324)	11.0	456.284 (456.281)	8.2
**P2-Val *^am^***	VSQVY*PIVQ	1031.584 (1031.588)	13.2	595.308 (595.309)	8.3	455.300 (455.298)	6.7
**P3-Gly *^am^***	VSGNY*PIVQ	975.529 (975.526)	11.1	539.248 (539.246)	6.0	455.300 (455.298)	6.6
**P3-Lys *^am^***	VSKNY*PIVQ	1046.602 (1046.599)	8.7	610.322 (610.320)	4.2	455.302 (455.298)	6.5

## Supplementary Materials

The following are available online at https://www.mdpi.com/2218-273X/10/7/1004/s1, Figure S1: Secondary structure prediction of SASP14 by different methods. Figure S2: Structures of Ddi1 and Ddi2 proteases as templates for modeling of SASP14. Figure S3: Model evaluation by ProSA web server. Figure S4: Hydrophobicity and residue volume profiles of cleavage site sequences. Figure S5: Putative hydrogen bonds between SASP14 and P3-Lys residue of the modified substrates. Figure S6: Phosphorylation of P4 residue prevents processing at filaggrin cleavage site. Figure S7: Unedited, original gel images. Table S1: Oligonucleotide primers used for cloning and mutagenesis. Table S2: Compositions of S2 and S3 substrate-binding cavities of HIV-1 and SASP14 proteases.
