# Supplementary material for: Biochemical Characterization of Human Retroviral-Like Aspartic Protease 1 (ASPRV1)
Source: Biomolecules. 2020 Jul 6;10(7):1004. doi: 10.3390/biom10071004 (PMC7408472; doi:10.3390/biom10071004)
Supplement: Supplementary file 1 [file biomolecules-10-01004-s001.zip › Figure S7.pdf]

Biochemical characterization of human retroviral-like aspartic protease 1 (ASPRV1)

Mária Golda, János A. Mótyán, Katalin Nagy,
Krisztina Matúz, Tibor Nagy, József Tőzsér

Unedited, original gel images

Comment to the figures:

Unedited, raw SDS-PAGE images are represented.

Boxes indicate those parts of the original images which were used to generate manuscript figures.

Samples are listed in the tables, where X denotes those non-relevant samples which are not shown in final images.

Figure 3.
Unedited, original image of the Coomassie-stained gel. Image was taken by using AlphaImager HP system (Protein Simple, USA). The part of the image that was used to generate manuscript figure is framed.

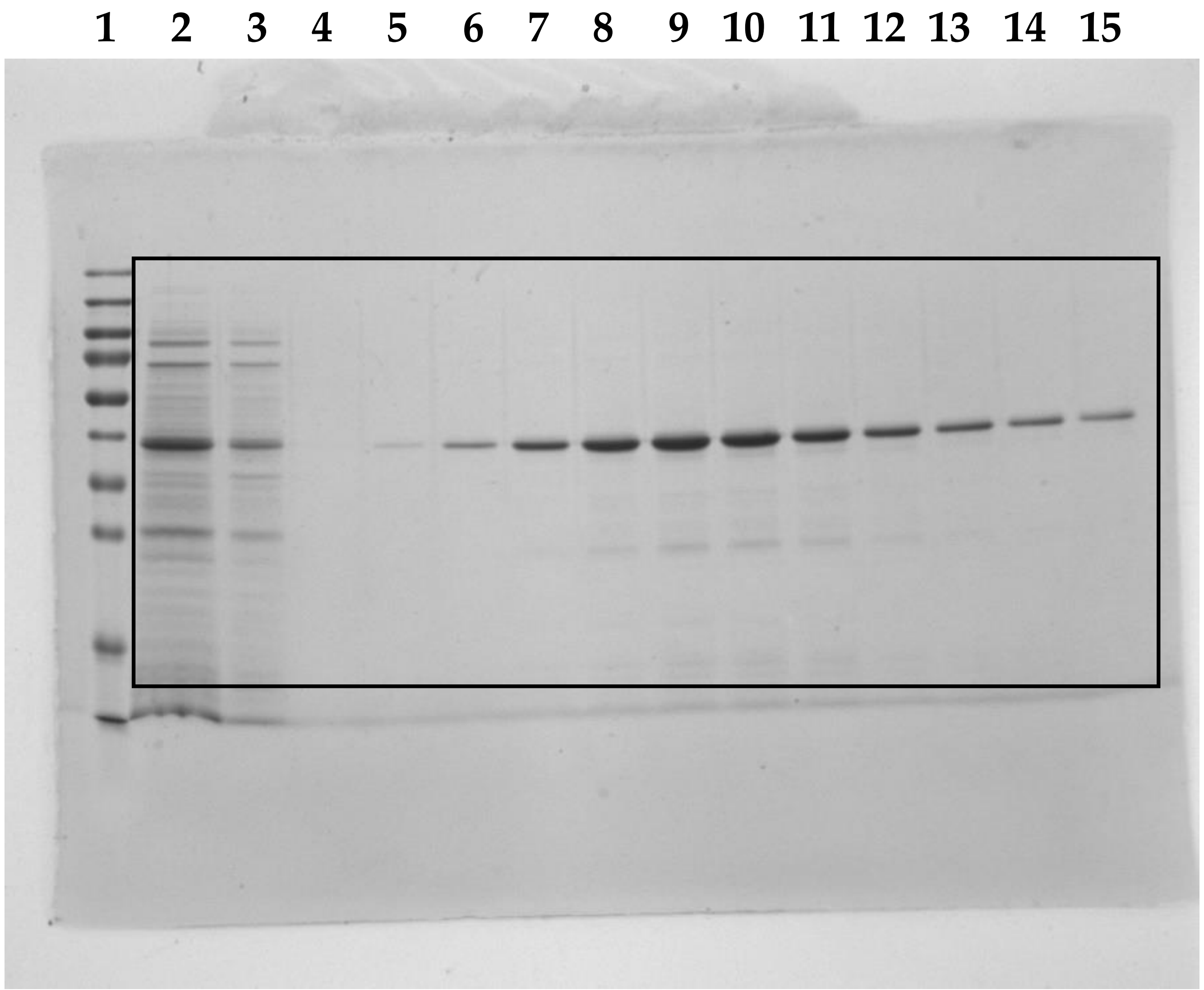

<u>Sample</u>

1. **Molecular weight standard**
   (PageRuler Prestained, #26616)
2. **Soluble lysate**
3. **Flow-through**
4. **Eluate fraction I.**
5. **Eluate fraction II.**
6. **Eluate fraction III.**
7. **Eluate fraction IV.**
8. **Eluate fraction V.**
9. **Eluate fraction VI.**
10. **Eluate fraction VII.**
11. **Eluate fraction VIII.**
12. **Eluate fraction IX.**
13. **Eluate fraction X.**
14. **Eluate fraction XI.**
15. **Eluate fraction XII.**

Figure 10a.
Unedited, original image of the Coomassie-stained gel. Image was taken by using AlphaImager HP system (Protein Simple, USA). The part of the image that was used to generate manuscript figure is framed.

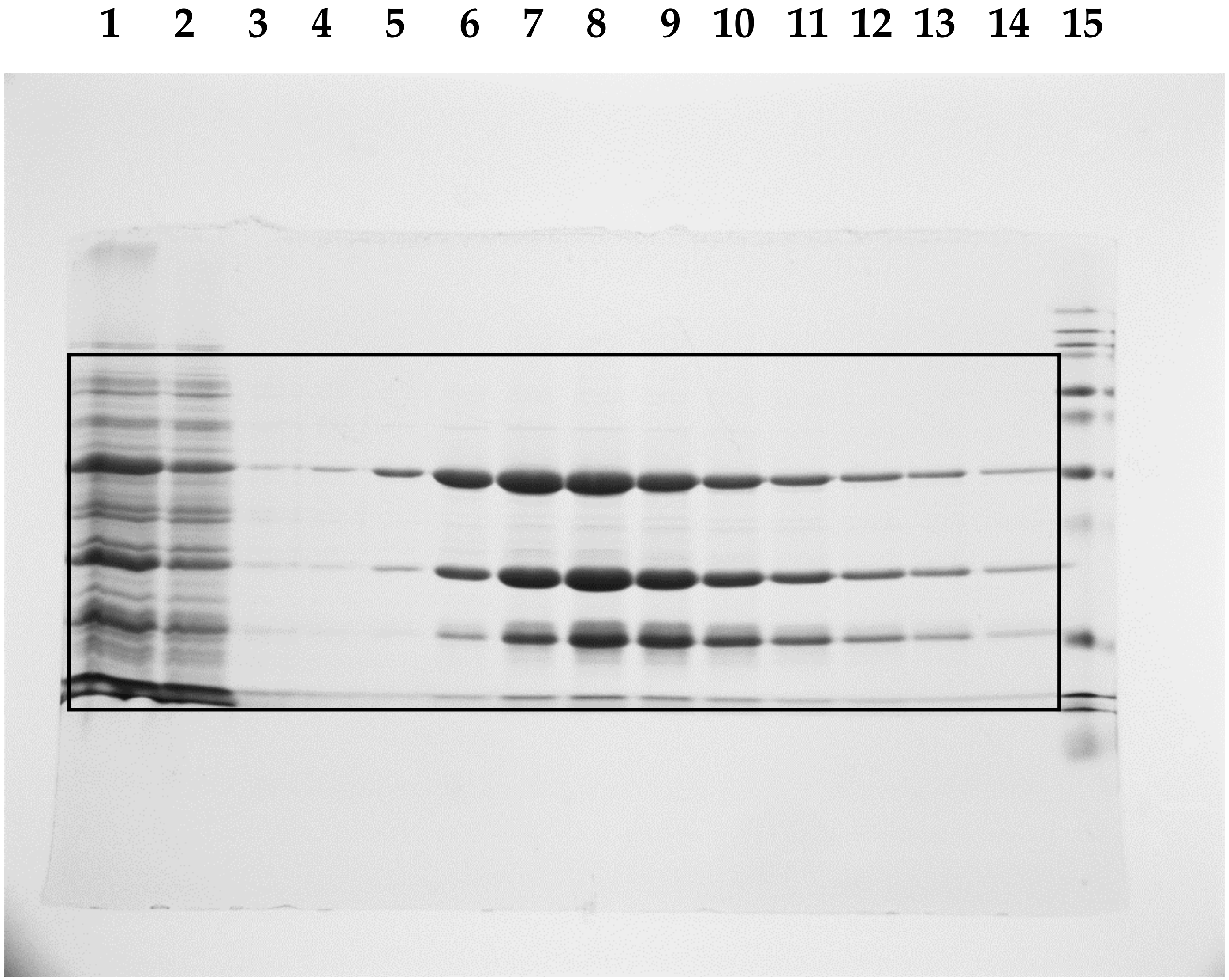

Sample

1. Soluble lysate

2. Flow-through

3. Eluate fraction I.

4. Eluate fraction II.

5. Eluate fraction III.

6. Eluate fraction IV.

7. Eluate fraction V.

8. Eluate fraction VI.

9. Eluate fraction VII.

10. Eluate fraction VIII.

11. Eluate fraction IX.

12. Eluate fraction X.

13. Eluate fraction XI.

14. Eluate fraction XII.

15. Molecular weight standard
(ProSieve QuadColor, Lonza Cat. No. 00193837)

Figure 10b.
Unedited, original image of the Coomassie-stained gel. Image was taken by using AlphaImager HP system (Protein Simple, USA). The part of the image that was used to generate manuscript figure is framed.

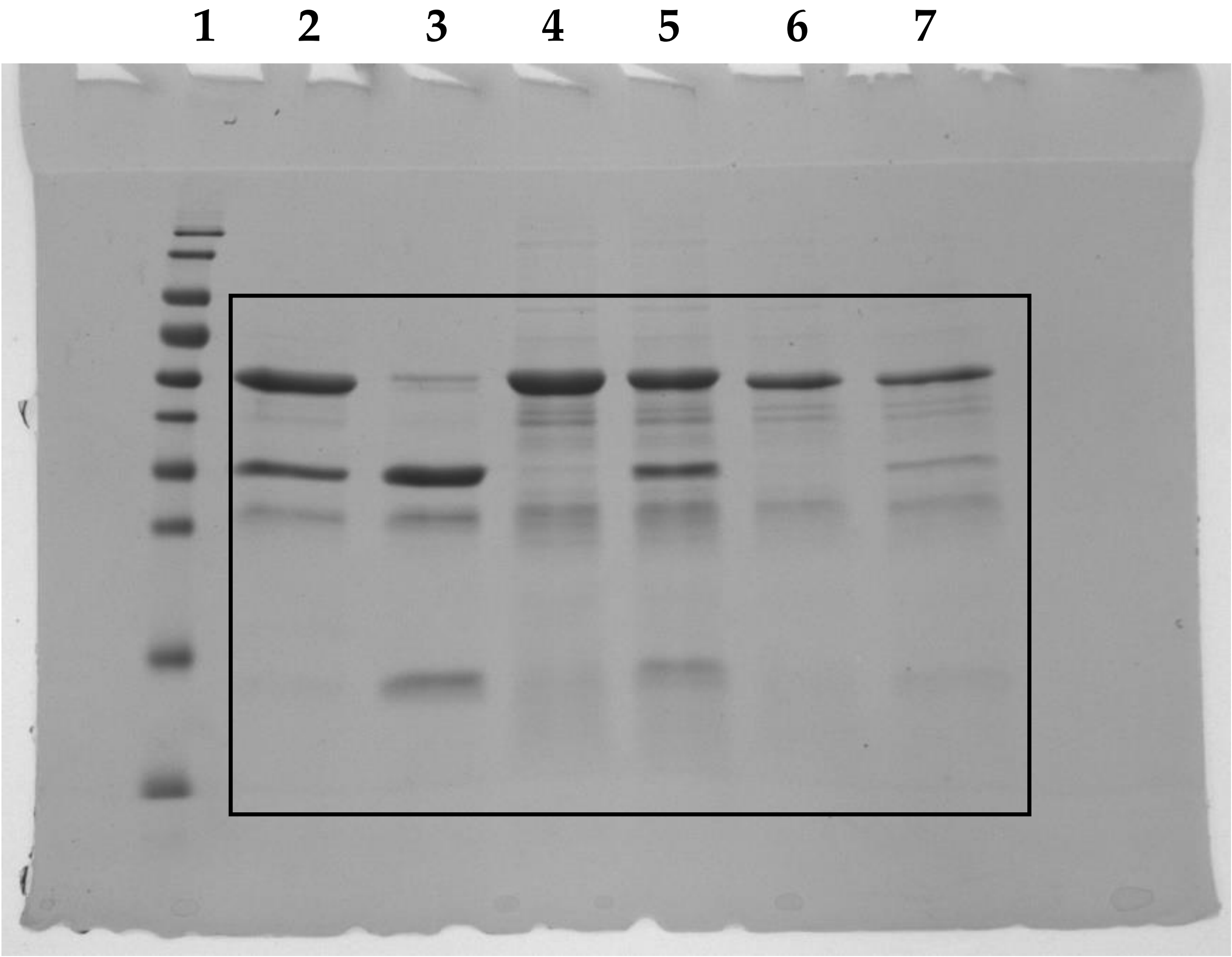

<u>Sample</u>

1. **Molecular weight standard** (PageRuler Prestained, #26616)
2. **GST-SASP28 wild-type 0h incubation time**
3. **GST-SASP28 wild-type 20h incubation time**
4. **GST-SASP28 A189K/N190I 0h incubation time**
5. **GST-SASP28 A189K/N190I 20h incubation time**
6. **GST-SASP28 A167G/L168G/A189K/N190I 0h incubation time**
7. **GST-SASP28 A167G/L168G/A189K/N190I 20h incubation time**

Figure 11a.
Unedited, original image of the Coomassie-stained gel. Image was taken by using AlphaImager HP system (Protein Simple, USA). The part of the image that was used to generate manuscript figure is framed.

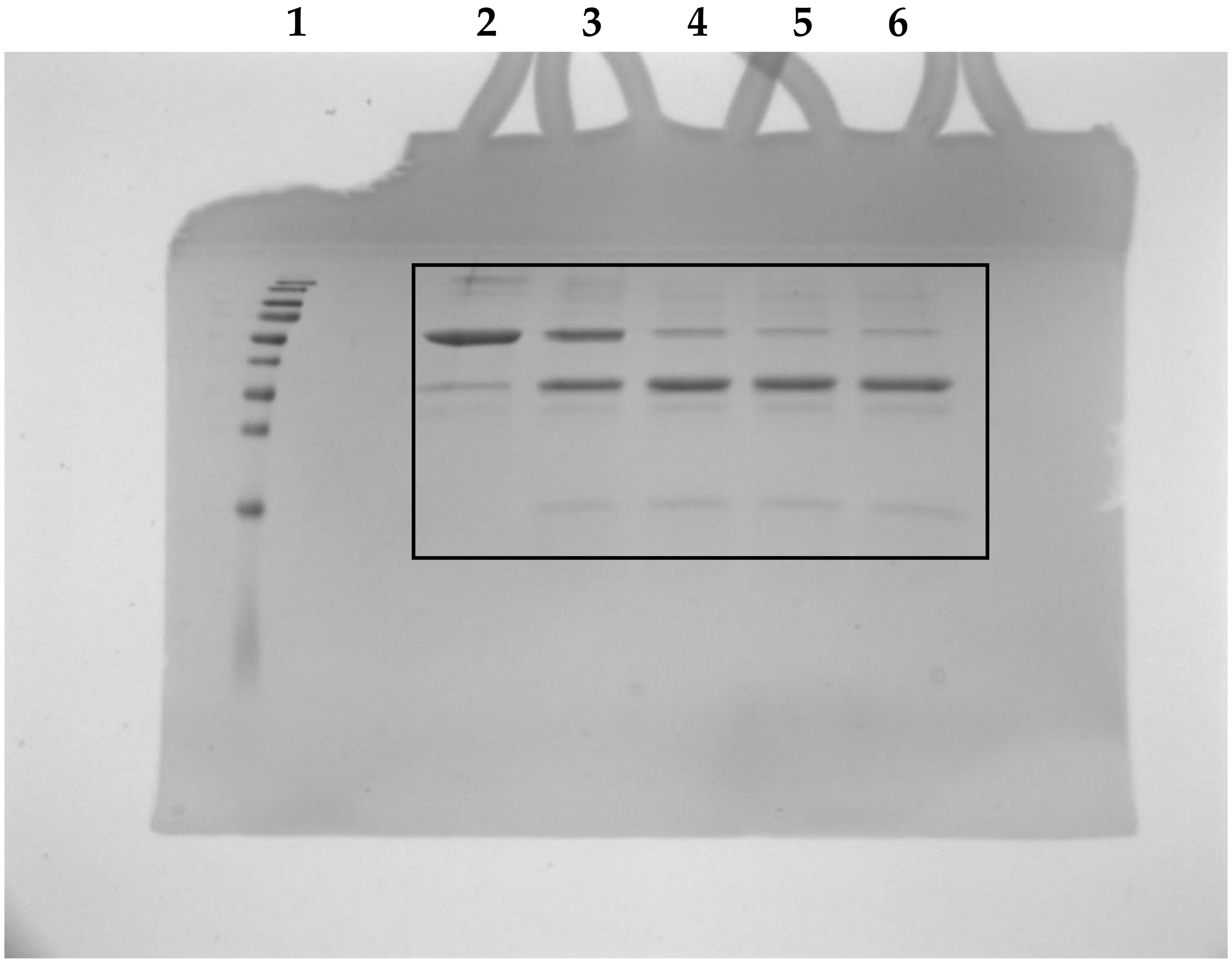

Sample

1. **Molecular weight standard** (PageRuler Prestained, #26616)
2. **GST-SASP28 wild-type 0 min incubation time**
3. **GST-SASP28 wild-type 5 min incubation time**
4. **GST-SASP28 wild-type 15 min incubation time**
5. **GST-SASP28 wild-type 30 min incubation time**
6. **GST-SASP28 wild-type 60 min incubation time**
