# Supplementary material for: Biochemical Characterization of Human Retroviral-Like Aspartic Protease 1 (ASPRV1)
Source: Biomolecules. 2020 Jul 6;10(7):1004. doi: 10.3390/biom10071004 (PMC7408472; doi:10.3390/biom10071004)
Supplement: Supplementary file 1 [file biomolecules-10-01004-s001.zip › Figure_S4.pdf]

Figure S4. Hydrophobicity and residue volume profiles of cleavage site sequences. (**a**) Hydrophobicity profiles were determined for natural cleavage sequences of ASPRV1 and HIV-1 proteases, based on hydropathy index values of residues. (**b**) Residue volumes were determined as it was described previously [24,25]. Average residue volumes are shown for ASPRV1 and HIV-1 protease cleavage site sequences by white and black columns, respectively. (**c**) List of cleavage site sequences used for the comparison of hydrophobicity profiles and residue volumes.

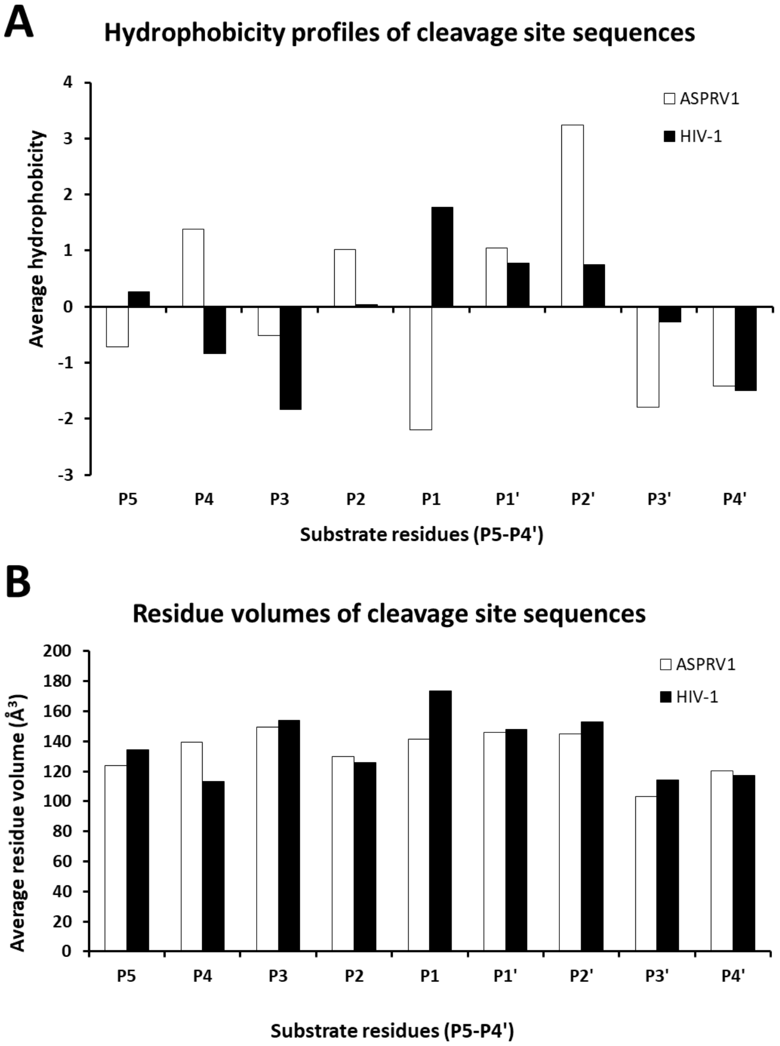

C

ASPRV1		HIV-1	
**SASP37/SASP28**	EESSR↓MAGSG	**MA/CA**	VSQNY↓PIVQ
**SASP28/SASP14**	IVFAN↓SMGKG	**CA/p2**	KARVL↓AEAM
**SASP14C-TERM**	EFDLE↓LIEED	**TF/PR**	VSFNF↓PQIT
**ALTERNATIVE**	TVKEA↓LLKAF	**PR/RT**	CTLNF↓PISP
**FILAGGRIN**	GSFLY↓QVSTH	**RT/IN**	IRKIL↓FLDG
		**p2/NC**	TATIM↓MQRG
		**NC/p1**	ERQAN↓FLGK
		**p1/p6**	RPQNF↓LQSR
		**RT/RH**	GAETF↓YVDG
