# Supplementary material for: Biochemical Characterization of Human Retroviral-Like Aspartic Protease 1 (ASPRV1)
Source: Biomolecules. 2020 Jul 6;10(7):1004. doi: 10.3390/biom10071004 (PMC7408472; doi:10.3390/biom10071004)
Supplement: Supplementary file 1 [file biomolecules-10-01004-s001.zip › Table_S2.pdf]

Table S2. Compositions of S2 and S3 substrate-binding cavities of HIV-1 and SASP14 proteases. Binding site-forming residues of HIV-1 and SASP14 proteases are shown. Bold letters indicate residues where side chain-side chain interaction may occur. Resides were classified as follows. Hydrophobic: A, V, I, L, F, W, C, M, P, G; hydrophilic: S, T, N, Q, Y, H; and charged: D, E, K, R. * Binding site-forming residues of HIV-1 PR have been identified previously [24, 25].

**S2 site**			**S3 site**	
**HIV-1***	**SASP14**		**HIV-1***	**SASP14**
**A28**	**A25**		**L10**	**Y7**
**D30**	**V27**		**R8**	**G5**
**V32**	**V29**		**D29**	**Q26**
**I47**	**V53**		**G48**	**K54**
G48	K54		**V82**	**E90**
G49	V55			
**I50**	**A56**			
**L76**	**L83**			
**I84**	**I93**			
86	100	hydrophobic (%)	60	20
0	0	hydrophylic (%)	0	40
14	0	charged (%)	40	40
