# Supplementary material for: Biochemical Characterization of Human Retroviral-Like Aspartic Protease 1 (ASPRV1)
Source: Biomolecules. 2020 Jul 6;10(7):1004. doi: 10.3390/biom10071004 (PMC7408472; doi:10.3390/biom10071004)
Supplement: Supplementary file 1 [file biomolecules-10-01004-s001.zip › Figure_S1.pdf]

Figure S1. Secondary structure prediction of SASP14 by different methods. Secondary structural organization of wild-type SASP14 was predicted by different methods, secondary structural elements are shown by different color: yellow, β-strand; red, α-helix; active site motif residues, black and bold. The additional helical insert was predicted by PredictProtein, JPred4 and SOPMA prediction algorithms, while the consensus helix and three C-terminal β-sheets were predicted by all methods.

```
               ....|....|....|...|....|....|....|....|....|....|....|....|....|....
PredictProtein SMGKGYYLKGKIGKVPVRFLVDSGAQVSVVHPNLWEEVTDGDLDTLQPFENVVKVANGAEMKILGVWD
JPred4         SMGKGYYLKGKIGKVPVRFLVDSGAQVSVVHPNLWEEVTDGDLDTLQPFENVVKVANGAEMKILGVWD
DSC            SMGKGYYLKGKIGKVPVRFLVDSGAQVSVVHPNLWEEVTDGDLDTLQPFENVVKVANGAEMKILGVWD
SOPMA          SMGKGYYLKGKIGKVPVRFLVDSGAQVSVVHPNLWEEVTDGDLDTLQPFENVVKVANGAEMKILGVWD
GOR4           SMGKGYYLKGKIGKVPVRFLVDSGAQVSVVHPNLWEEVTDGDLDTLQPFENVVKVANGAEMKILGVWD

               |....|....|...|....|....|....|....|....|....|....|....|....|....|...
PredictProtein TAVSLGKLKLKAQFLVANASAEEAIIGTDVLQDHNAILDFEHRTCTLKGKKFRLLPVGGSLEDEFDLE
JPred4         TAVSLGKLKLKAQFLVANASAEEAIIGTDVLQDHNAILDFEHRTCTLKGKKFRLLPVGGSLEDEFDLE
DSC            TAVSLGKLKLKAQFLVANASAEEAIIGTDVLQDHNAILDFEHRTCTLKGKKFRLLPVGGSLEDEFDLE
SOPMA          TAVSLGKLKLKAQFLVANASAEEAIIGTDVLQDHNAILDFEHRTCTLKGKKFRLLPVGGSLEDEFDLE
GOR4           TAVSLGKLKLKAQFLVANASAEEAIIGTDVLQDHNAILDFEHRTCTLKGKKFRLLPVGGSLEDEFDLE
```
