# Supplementary material for: Biochemical Characterization of Human Retroviral-Like Aspartic Protease 1 (ASPRV1)
Source: Biomolecules. 2020 Jul 6;10(7):1004. doi: 10.3390/biom10071004 (PMC7408472; doi:10.3390/biom10071004)

Figure S6. Phosphorylation of P4 residue prevents processing at filaggrin cleavage site. Representative chromatograms are shown for cleavage reactions. Synthetic oligopeptide substrates representing the P2-Leu mutant HIV-1 MA/CA cleavage site (VSQLY↓PIVQ) (**a**), the cleavage site of SASP14 in pro-FLG (GSFLY↓QVSTH) (**b**), and the P4-Ser phosphorylated variant of pro-FLG cleavage site (**c**) were applied. Arrows indicate substrates and cleavage products: S, substrate; $_{P}$S, phosphorylated substrate; P1, product 1; P2, product 2. Samples shown in the chromatograms are labelled: substrate control, red; enzyme control, black; enzyme+substrate, green.

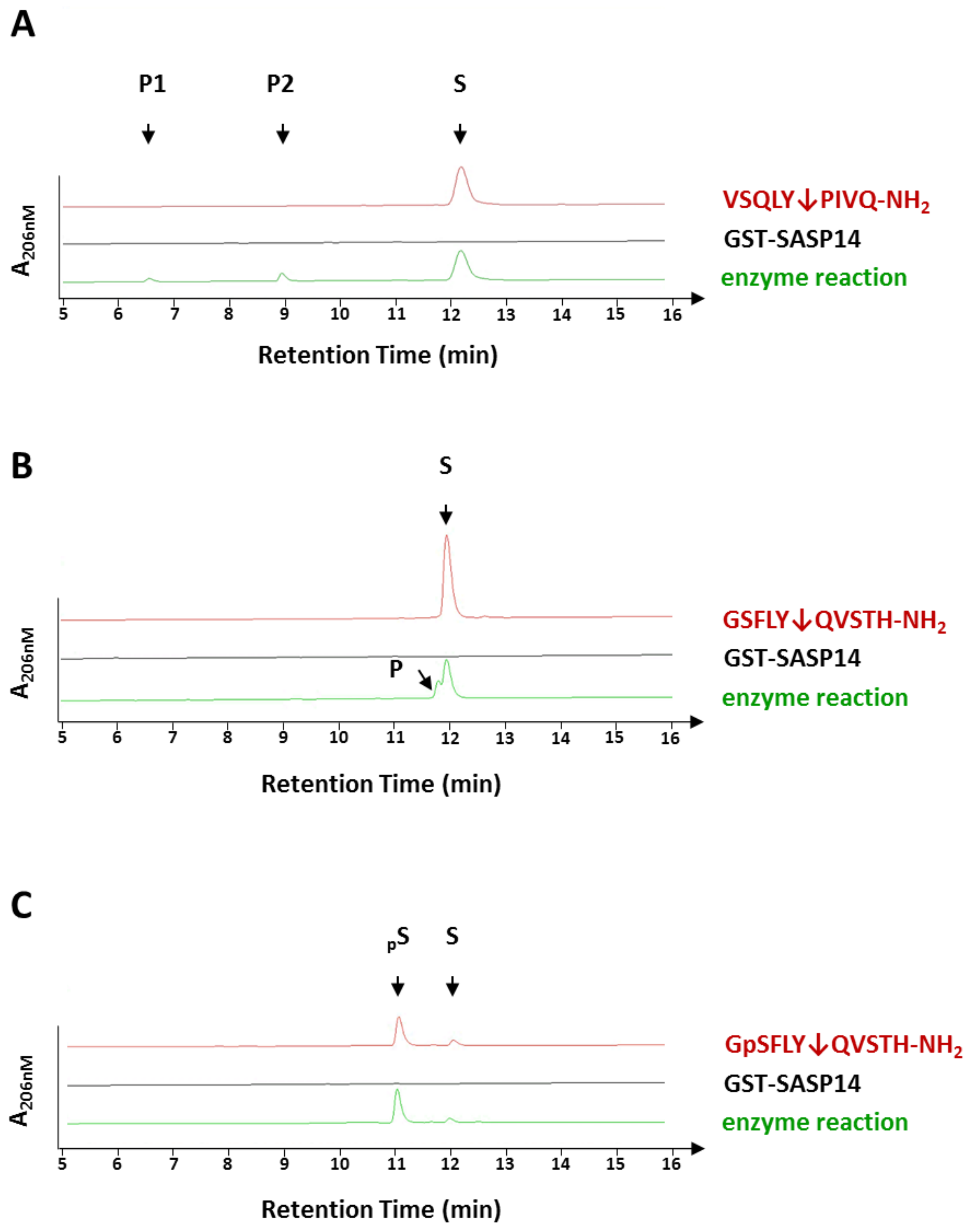

Supplement: Supplementary file 1 [file biomolecules-10-01004-s001.zip › Figure_S6.pdf]
