# Supplementary material for: Biochemical Characterization of Human Retroviral-Like Aspartic Protease 1 (ASPRV1)
Source: Biomolecules. 2020 Jul 6;10(7):1004. doi: 10.3390/biom10071004 (PMC7408472; doi:10.3390/biom10071004)

Figure S5. Putative hydrogen bonds between SASP14 and P3-Lys residue of the modified substrates. Figure shows proposed model of SASP14 complexed with P3-Lys modified version of VSQNY↓PIVQ oligopeptide. Hydrogen bonds are shown by dashed grey lines. Color code: yellow, β-strand; red, α-helix; green, loop.

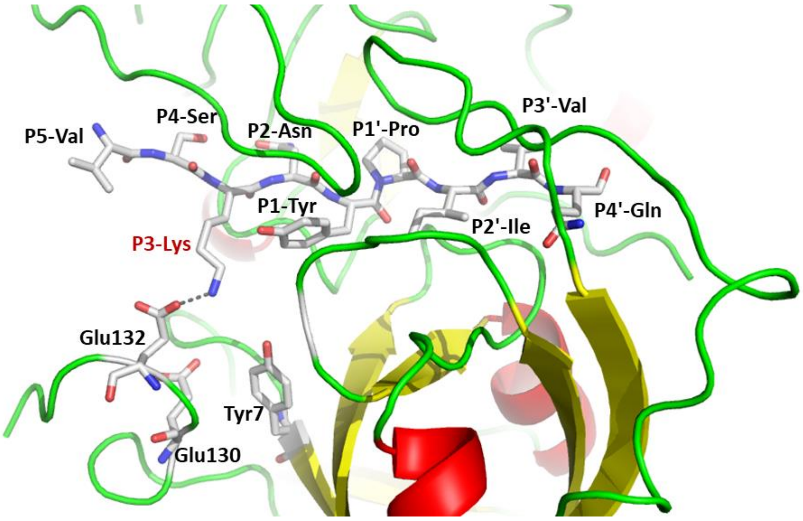

Supplement: Supplementary file 1 [file biomolecules-10-01004-s001.zip › Figure_S5.pdf]
