# Supplementary material for: Biochemical Characterization of Human Retroviral-Like Aspartic Protease 1 (ASPRV1)
Source: Biomolecules. 2020 Jul 6;10(7):1004. doi: 10.3390/biom10071004 (PMC7408472; doi:10.3390/biom10071004)

Figure S3. Model evaluation by ProSA web server. Homology model of SASP14 and Ddi1 template structures were analyzed by ProSA web server. z-score indicates overall model quality, while local model quality is shown by plotting energies as a function of amino acid sequence position (positive values correspond to problematic parts of the input structure).

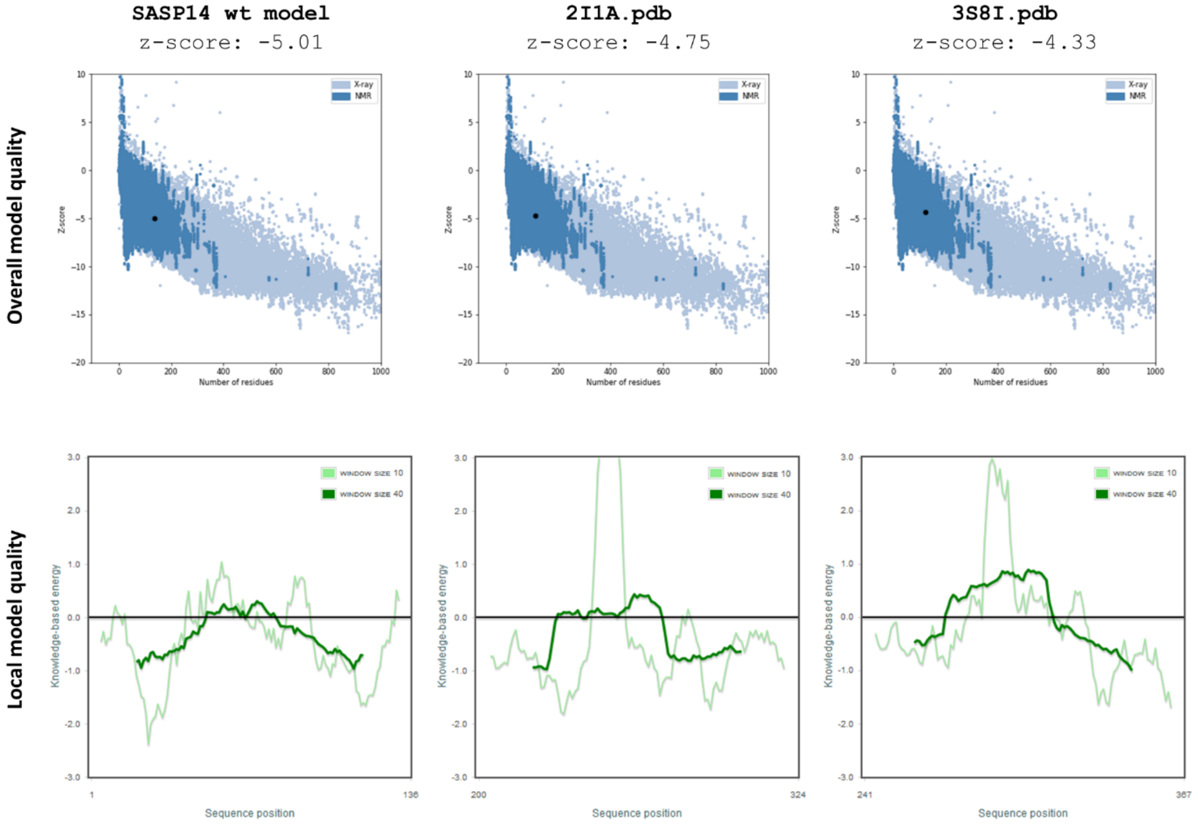

Supplement: Supplementary file 1 [file biomolecules-10-01004-s001.zip › Figure_S3.pdf]
