# Supplementary material for: Biochemical Characterization of Human Retroviral-Like Aspartic Protease 1 (ASPRV1)
Source: Biomolecules. 2020 Jul 6;10(7):1004. doi: 10.3390/biom10071004 (PMC7408472; doi:10.3390/biom10071004)
Supplement: Supplementary file 1 [file biomolecules-10-01004-s001.zip › Figure_S2.pdf]

Figure S2. Structures of Ddi1 and Ddi2 proteases as templates for modeling of SASP14. (**a**) Template search by SWISS-MODEL resulted in Ddi1 and Ddi2 proteases as potential templates for modeling of SASP14, the obtained data are shown. (**b**) Structure alignment of Ddi1 and Ddi2 protease structures by mTm-Align web server, the PDB identifiers of templates are shown. Common core regions are shown by magenta color. RMSD values (Å) obtained from the comparison are shown below the aligned sequences. (**c**) Comparison of SASP14 homology model and structures of Ddi1 and Ddi2 proteases. Ddi proteases contain no closed conformational flaps.

A

Name	Title	GMQE	QSQE	Seq identity	Range	Coverage	Method	Oligo State	Ligands
5ys4.2.A	DNA-damage inducible protein DDI1-like protein	0.50	0.26	27.12	4-124	0.87	X-ray, 2.30 Å	homo-dimer	None
5yq8.1.A	DNA-damage inducible protein DDI1-like protein	0.51	0.24	28.45	4-124	0.85	X-ray, 2.25 Å	homo-dimer	None
4rgh.1.A	Protein DDI1 homolog 2	0.51	0.23	23.08	5-125	0.86	X-ray, 1.90 Å	homo-dimer	None
3s8i.1.A	Protein DDI1 homolog 1	0.49	0.19	19.49	5-125	0.87	X-ray, 1.70 Å	homo-dimer	None
2i1a.2.A	DNA damage-inducible protein DDI1	0.46	0.20	19.13	5-124	0.85	X-ray, 2.30 Å	homo-dimer	None
4z2z.1.A	DNA damage-inducible protein 1	0.45	0.18	19.83	5-124	0.85	X-ray, 1.80 Å	homo-dimer	None

B

```
              10        20        30        40        50        60        70        80        90       100       110       120       130       140
     .........|.........|.........|.........|.........|.........|.........|.........|.........|.........|.........|.........|.........|.........|....
2I1A -----------QVPMLYINIEINNYPVKAFVDTGAQTTIMSTRLAKKTGLSRMIDKRF------------IIGRIHQAQVKIETQYIPCSFTVLD-TDIDVLIGLDMLKRHLACVDLKENVLRI-AE-VETSFLSEAEIP---
3S8I ------------VTMLYINCKVNGHPLKAFVDSGAQMTIMSQACAERCNIMRLVDRRWAGVA---KG-QRIIGRVHLAQIQIEGDFLQCSFSILEDQPMDMLLGLDMLRRHQCSIDLKKNVLVIGTTGTQTYFLPEGELP---
4RGH -----------QVVMLYINCKVNGHPVKAFVDSGAQMTIMSQACAERCNIMRLVDRRWAGIAKG-VGTQKIIGRVHLAQVQIEGDFLPCSFSILEEQPMDMLLGLDMLKRHQCSIDLKKNVLVIGTTGSQTTFLPEGELP---
4Z2Z NAIEYTPE--TQVPMLYINIEINNYPVKAFVDTGAQTTIMSTRLAKKTGLSRMIDKRF------------IIGRIHQAQVKIETQYIPCSFTVLD-TDIDVLIGLDMLKRHLACVDLKENVLRI-AE-VETSFLSEAEIP---
5YQ8 ----------AKVTMLYVPCTINQVLVKAFVDSGAQNSIMNKRTAERCGLMRLVDVRMRGVAVGVGR-QEICGRIHMTPVNLAGMYIPFAFYVIEDQAMDLIIGLDQLKRHQMMIDLKHNCLTI--DNINVPFLPENDLPALA
5YS4 --------AFAKVTMLYVPCTINQVLVKAFVDSGAQNSIMNKRTAERCGLMRLVDVRM----------QEICGRIHMTPVNLAGMYIPFAFYVIEDQAMDLIIGLDQLKRHQMMIDLKHNCLTI--DNINVPFLPENDLP---
```

RMSD	2i1a	3s8i	4rgh	4z2z	5yq8	5ys4
2i1a	0.000	0.797	0.824	0.449	0.985	1.058
3s8i		0.000	0.831	0.820	1.129	0.885
4rgh			0.000	0.861	1.086	0.795
4z2z				0.000	1.084	1.391
5yq8					0.000	0.879
5ys4						0.000

C

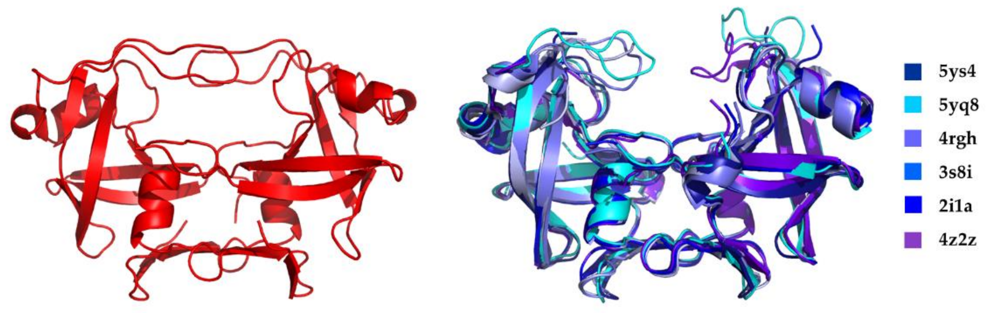

Homology model of SASP14

Structures of Ddi1 and Ddi2 proteins
