# Supplementary material for: Biochemical Characterization of Human Retroviral-Like Aspartic Protease 1 (ASPRV1)
Source: Biomolecules. 2020 Jul 6;10(7):1004. doi: 10.3390/biom10071004 (PMC7408472; doi:10.3390/biom10071004)
Supplement: Supplementary file 1 [file biomolecules-10-01004-s001.zip › Table_S1.pdf]

Table S1. Oligonucleotide primers used for cloning and mutagenesis. (**a**) The oligonucleotide primers used to amplify SASP37, SASP28, and SASP14 sequences from pCMV6-XL4-asprv1 vector. Codon encoding Ile2 residue is underlined in forward-BamH1-SASP14 primer (wild-type residue is Met2 according to SASP14 numbering). (**b**) Oligonucleotide primers used for mutagenesis are listed here and were deposited to the public oligonucleotide database of Laboratory of Retroviral Biochemistry: http://lrb.med.unideb.hu/research/oligos.

A)

**Name**	**Sequence**
forward-BamH1-SASP37	5′-G ATA GGA TCC GGG AGC CCA GGG GC-3′
forward-BamH1-SASP28	5′-G ATA GGA TCC GCC GGG AGC GGA GCC-3′
forward-BamH1-SASP14	5′-G ATA GGA TCC AGC **ATC** GGT AAG GGC-3′
reverse-EcoR1-SASP37/28	5′-CT ATT GAA TTC TCA GTG GGA TAG CTC CTG CCG-3′
reverse-EcoR1-SASP14	5′-CT ATT GAA TTC TCA CTC CAG GTC AAA CTC ATC TTC CAG-3′

B)

**Name**	**Sequence**
Processing site mutant: SASP28-A189K/N190I	
SASP28-A189K/N190I	5′-CTG CCC AAA GAG ATC GTC TTT AAG ATC AGC ATG GGT AAG GGC TAC-3′
Processing site mutant: SASP28-A167G/L168G/A189K/N190I	
SASP28-A189K/N190I	5′-CTG CCC AAA GAG ATC GTC TTT AAG ATC AGC ATG GGT AAG GGC TAC-3′
SASP28-A167G/L168G	5′-G GG ACT GTG AAA GAG GGC GGC CTG AAG GCC TTT GGG-3′
